# ADAR1 expression is associated with cervical cancer progression and negatively regulates NK cell activity

**DOI:** 10.1172/jci.insight.190244

**Published:** 2025-07-08

**Authors:** Valentina Tassinari, Marta Kaciulis, Stefano Petrai, Helena Stabile, Angelina Pernazza, Martina Leopizzi, Valeria Di Maio, Francesca Belleudi, Danilo Ranieri, Vanessa Mancini, Innocenza Palaia, Federica Tanzi, Ludovica Lospinoso Severini, Silvia Ruggeri, Maria Emanuela Greco, Giovanni Bernardini, Alessandra Zingoni, Marco Cippitelli, Cristina Cerboni, Alessandra Soriani

**Affiliations:** 1 Department of Molecular Medicine,; 2 Department of Medical-Surgical Sciences and Biotechnologies, and; 3 Department of Clinical and Molecular Medicine, Sapienza University of Rome, Rome, Italy.; 4 Department of Life Sciences, Health and Professions, Link Campus University, Rome, Italy.; 5 Department of Maternal and Child Health and Urological Sciences and; 6 Pasteur Institute–Cenci Bolognetti Foundation, Sapienza University of Rome, Rome, Italy.

**Keywords:** Immunology, Inflammation, Oncology, Cervical cancer, Innate immunity, NK cells

## Abstract

ADAR1 edits double-stranded RNAs (dsRNAs) by deaminating adenosines into inosines, preventing aberrant activation of innate immunity by endogenous dsRNAs, which may resemble viral structures. Several tumors exploit ADAR1 to evade immune surveillance; indeed, its deletion reduces tumor viability and reshapes infiltrating leukocytes. Here we investigated the role of ADAR1 in immune evasion mechanisms during cervical cancer (CC) progression. Patients’ biopsy samples showed higher ADAR1 expression already in premalignant lesions (squamous intraepithelial lesions [SIL]) and a substantially reduced percentage of infiltrating CD7^+^ innate cells in in situ and invasive carcinomas compared with normal mucosa, with CD56^+^ NK cells showing phenotypic alterations that may have affected their functional responses. In CC-derived cell lines (SiHa, CaSki), ADAR1 silencing reduced cell proliferation, an effect further enhanced by exogenous IFN-β administration. It also induced proinflammatory gene expression, as demonstrated by RNA-Seq analysis, and conditioned supernatants collected from these cells activated several NK cell effector functions. NK cell infiltration and activation were also confirmed in organotypic 3D tissue models of SiHa cells knocked out for ADAR1. In conclusion, ADAR1 expression increased with CC progression and was accompanied by alterations in tumor-infiltrating NK cells, but its silencing in CC-derived cell lines potentiated antitumor NK cell activities. Thus, ADAR1 inhibition may represent a therapeutic perspective for CC and possibly other malignancies.

## Abstract 2

<span>Targeting ADAR1 in cervical cancer suppresses proliferation and induces proinflammatory factors, promoting NK cell activation. This </span><span>might represent </span><span>an immunostimulatory approach beyond cervical cancer patients.</span>

## Introduction

Cervical cancer (CC) is the fourth leading cause of cancer-related mortality in women (1). It takes years or decades to develop, and it is distinguished by a spontaneous continuous progression, starting with a persistent intraepithelial human papillomavirus (HPV) infection in almost all cases, evolving to squamous intraepithelial lesions (SILs) and then into invading tumors and metastasis (2). Although prophylactic vaccines are available, a large portion of the population remains unvaccinated, and the vaccine does not prevent cancer development in individuals already exposed to the virus (3).

From an immunological perspective, CC is classified as an immune-infiltrated yet immunosuppressive malignancy. Despite the presence of an antitumor immune response, its effectiveness is often compromised during disease progression, primarily due to HPV-mediated modulation of the tumor microenvironment (TME) (3, 4).

Indeed, effective evasion of immune recognition seems to be the hallmark of HPV infections already at earlier stages, as the virus is almost invisible to the immune system due to an exclusively intraepithelial infectious cycle — with no viremic phase and no virus-induced cell death — and viral replication and release are not associated with inflammation. In addition, HPV downregulates innate immune signaling pathways; proinflammatory cytokines, particularly type I interferons (IFN-Is), are not released and the signals for antigen-presenting cell activation and recruitment are either not present or inadequate. This immune ignorance results in chronic infections that persist over weeks and months. Progression to high-grade SIL (HSIL) is associated with further deregulation of immunologically relevant molecules — particularly chemotactic chemokines and their receptors — on keratinocytes and endothelial cells of the underlying microvasculature, limiting or preventing infiltration of cytotoxic effectors into the lesion (5, 6).

NK cells are innate lymphocytes with a critical cytotoxic and immunoregulatory role, and although they are disseminated in the uterine mucosa, their role in the natural history of HPV infection and HPV-driven tumorigenesis is not entirely clear. It was reported that they emerge at an early stage in HPV-infected lesions and NK cells are present at higher level in premalignant lesions with a lower viral load (7). In studies of individuals with confirmed SILs of different grades, lesion regression strongly correlated with early infiltration of intraepithelial effector cytotoxic cells (granzymeB^+^CD8^+^ [GzmB^+^CD8^+^] and GzmB^+^CD56^+^) (8, 9). More recent studies applying single-cell multi-omics technologies provided new in-depth maps of the complex CC ecosystem (10–14). One common characteristic was the observation of an increased NK cell infiltrate in the tumor area, often (but not always) accompanied by an enriched cytotoxicity signature (e.g., GZMB, GZMH, PRF1), higher expression of genes involved in migration and adhesion, and lower levels of inhibitory molecules (e.g., TIGIT, CTLA-4). Moreover, expression of some of these genes was associated with a better prognosis (11–13), consistent with the observation that patients with CC with a high level of intratumoral NK cells had a decreased risk of progression (10). Interestingly, in one of these studies, the heterogeneity of malignant cells was investigated in relation to TME. Among the different cellular states uncovered, one was characterized by a bidirectional tumor stroma–immune system interaction involving NK and T cells through IFN signaling (14). However, to the best of our knowledge, beyond such transcriptomic approaches, other studies analyzing the phenotype of infiltrating NK cell subsets or of other innate lymphoid cells (ILCs) isolated from patients’ biopsy samples are lacking.

Over the last 15 years, several populations of ILCs have been described and classified into 5 groups (NK cells, ILC1, ILC2, ILC3, and LTi) according to their transcription factors (TFs) and cytokine production profile (15). In particular, NK cells are historically identified as CD56^+^CD16^+/–^CD127^–^EOMES^+^ (13). Although ILCs are now emerging as important players in numerous tumor types, their role has not been thoroughly investigated in either intraepithelial lesions or invasive carcinomas (ICs) of the cervix.

Antitumor responses are also dependent on IFNs, and loss of IFN signaling results in resistance to immune checkpoint therapies (16–19). Expression of IFN-I can be induced by long, fully base-paired dsRNAs deriving from viruses, but also by certain endogenous self RNAs that could aberrantly stimulate innate immune responses (20, 21). To prevent an erroneous activation of cytosolic sensors by self dsRNAs, ADAR1 — a member of the adenosine deaminase acting on RNA (ADAR) family of enzymes — edits such dsRNAs (20–22) by deaminating adenosines to inosines (A-to-I conversion), which are subsequently read out as guanosines, leading to transcriptomic and proteomic changes (20). Indeed, in many types of tumors, a substantial amount of mutational load is due to RNA editing/hyperediting by ADAR1 (21, 23). Moreover, recent studies demonstrate that ADAR1 deletion reduces cancer cell viability via IFN-I pathway activation (24, 25), and in ADAR1-null tumors, there is a global reshaping of immune cell profiles, suggesting that inflammation caused by ADAR1 deletion can bypass the loss of tumor-specific CD8^+^ T cells (19, 26). This evidence is in line with the fact that ADAR1 is considered a “master regulator” of cytoplasmic innate immunity, as it prevents autoimmunity (22). Indeed, in humans, loss-of-function mutations of ADAR1 can confer severe interferonopathies and autoimmune diseases (27).

There are few studies on the role of ADAR1 in HPV-driven tumorigenesis. An increase in expression was associated with CC progression (28, 29), and its ablation correlated with a proinflammatory phenotype (30), while a particular ADAR1 haplotype was related to recurrent dysplasia in patients coinfected with HPV/HIV (31). However, whether ADAR1 affects NK/ILC effector functions is not known, and to our knowledge, the interplay among ADAR1, IFN-I, and NK cells has not been previously addressed in any tumor model, including CC. Thus, in our study we investigated whether ADAR1 plays a role in CC tumorigenesis via its ability to dampen IFN-I signaling and production, with potential effects on NK/ILC-mediated innate immune responses as well.

## Results

### ADAR1 expression correlates with disease progression in CC.

**Figure 1:**
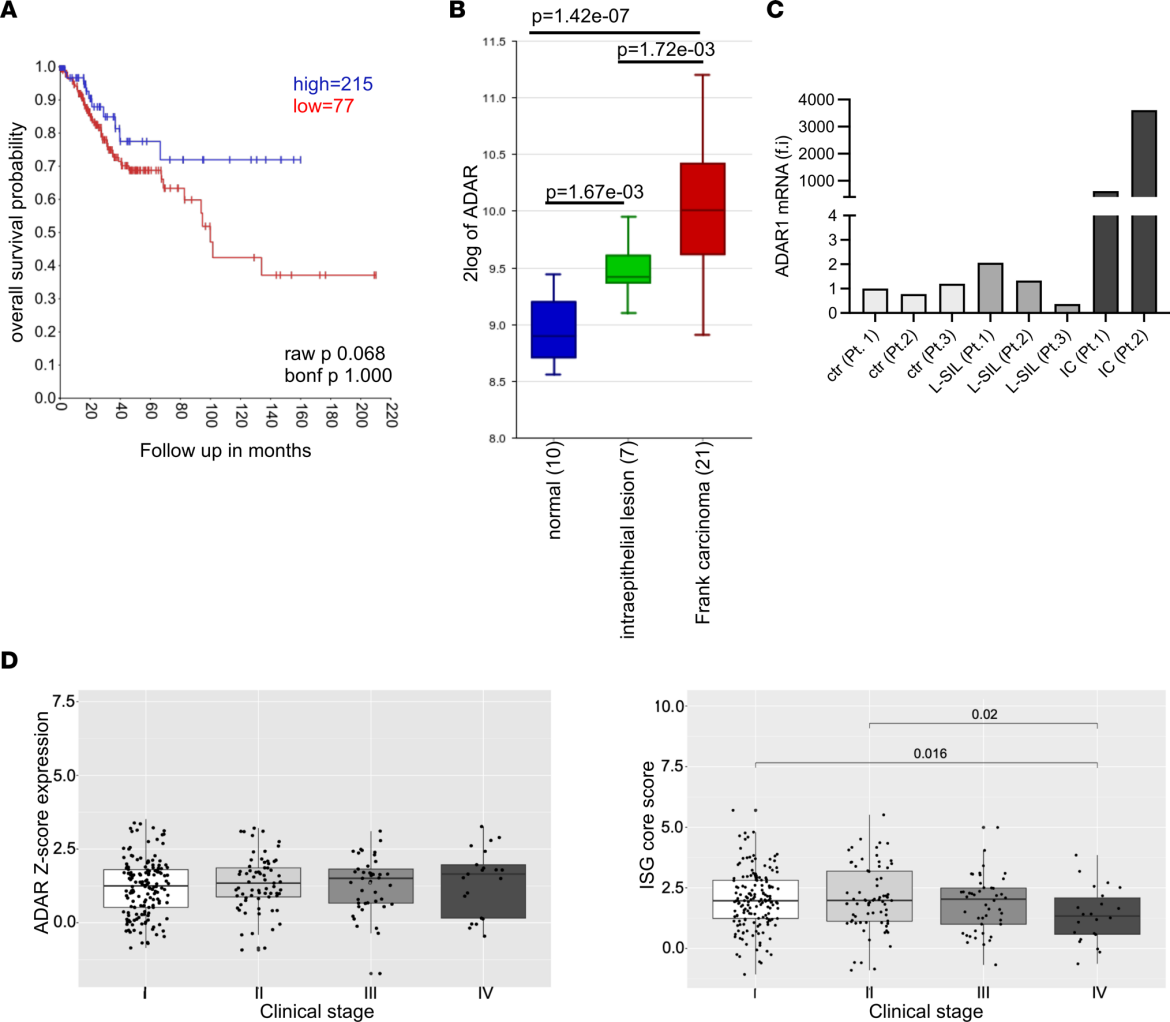
*ADAR1* overexpression characterizes CC progression. (**A**) Kaplan-Meier survival plot for CC patients (*n* = 292) stratified by low (red line) and high (blue line) *ADAR1* expression (cutoff mode: scan). Data were obtained from TCGA. For survival analysis, statistical significance was assessed with the log-rank test. (**B**) *ADAR1* expression was assayed on single HG_U133A arrays. Gene Expression Omnibus database (GEO GSE7803). (**C**) Total RNA was extracted from paraffin-embedded biopsy samples, and *ADAR1* expression was analyzed by RT-PCR. (**D**) *ADAR* expression (left panel) and *ISG* core score (right panel) at different clinical stages in the TCGA data. Between-group *P* values were computed using Wilcoxon’s rank-sum test. bonf, Bonferroni post hoc test; f.i, fold increase; Pt. patient Ctr, normal mucosa; IC, invasive CC.

To determine the importance of ADAR1 in the progression of CC, we first asked whether ADAR1 expression can represent a prognostic factor for patients’ survival. TCGA data revealed that high *ADAR1* levels were predictive of poor overall patient survival (Figure 1A), and R2: Genomics Analysis and Visualization Platform data also showed a significant progressive increase in *ADAR1* expression from normal tissues to SIL to IC (Figure 1B). These initial findings were confirmed by real-time PCR (RT-PCR) performed on mRNA extracted from formalin-fixed, paraffin-embedded tissues derived from normal mucosa, low-grade SIL (LSIL), or IC biopsies. Indeed, ADAR1 expression increased markedly during disease progression (Figure 1C). Additional analysis of TCGA data for *ADAR1* expression in ICs at different stages of the disease revealed a tendency toward increased *ADAR1* expression from stage I to stage IV, which was associated with a significant decrease in interferon-stimulated gene (*ISG*) expression in more advanced tumors (Figure 1D and Supplemental Table 1; supplemental material available online with this article; https://doi.org/10.1172/jci.insight.190244DS1).

**Figure 2:**
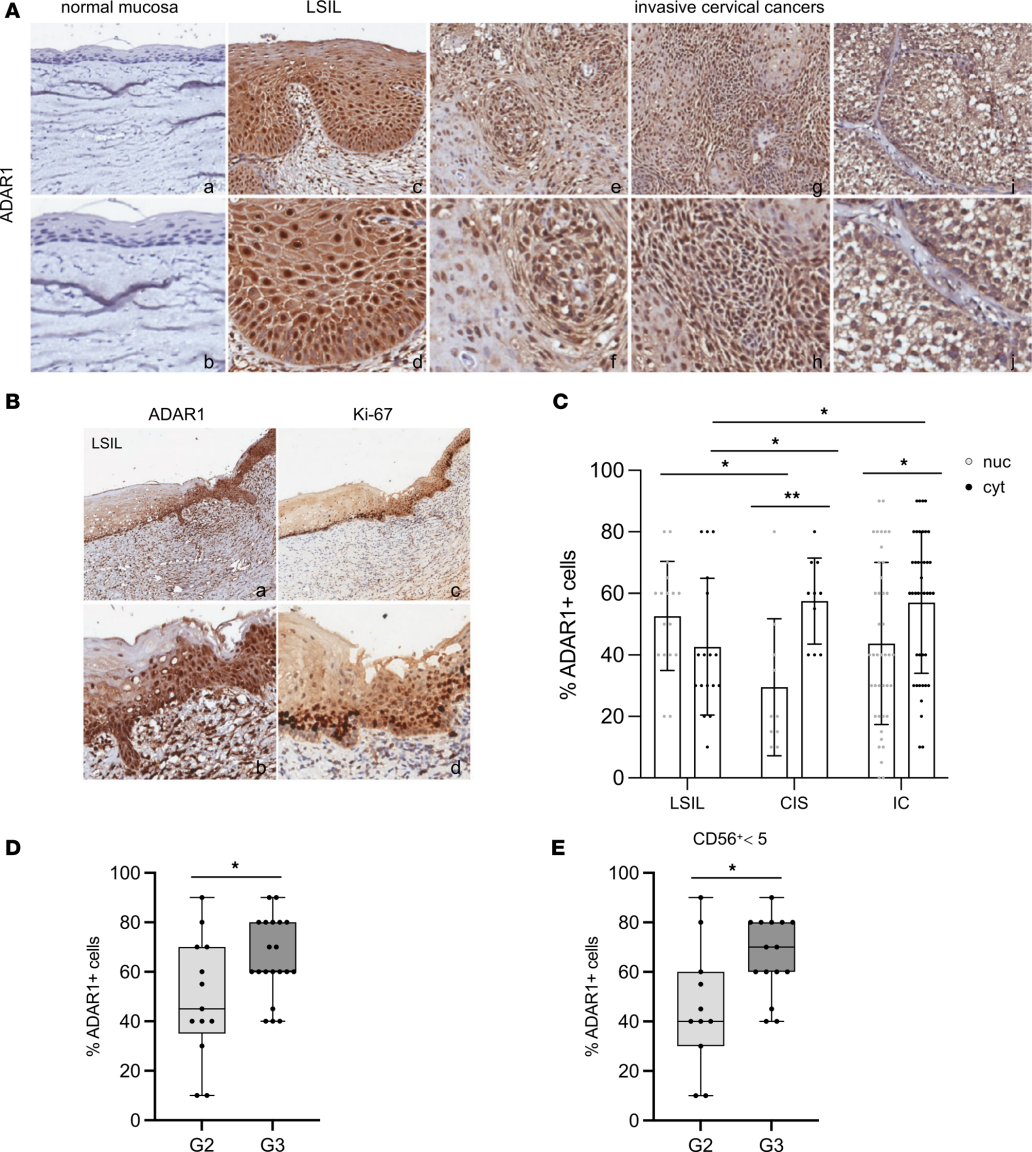
Expression of ADAR1 in normal cervical mucosa, premalignant lesions, and CCs. (**A**) Expression of ADAR1 on normal mucosa (immunostains; original magnification, 5× and 20×) (a and b), premalignant lesions (LSIL) (c and d), and invasive CCs (e–j) (immunostains; original magnification, 8× top row; 20× bottom row) was analyzed by IHC. (**B**) Representative images of IHC staining of ADAR1 compared with Ki-67 expression on LSIL lesions (immunostains; original magnification, 8× and 10×). (**C**) Quantification (%) of cytoplasmic (cyt) and nuclear (nuc) ADAR1 expression in the different lesions (17 LSIL, 10 HSIL/CIS, 41 IC). (**D**) Quantification (%) of total ADAR1 in G2–G3 IC stained sections. *P* values were calculated by ANOVA. (**E**) Expression of total ADAR1^+^ cells in G2 and G3 lesions with low numbers of infiltrating CD56^+^ cells (CD56^+^ <5). The *P* value was calculated by ANOVA. *P < 0.05, **P < 0.01.

Expression of ADAR1 in CC progression was further investigated by IHC on a panel of paraffin-embedded tissue samples (Figure 2). While ADAR1 was almost undetectable in normal mucosa (Figure 2A, a and b), its expression increased noticeably during disease progression, with clear positivity already in the LSIL (Figure 2A, panels c and d). Thus, we compared ADAR1 expression with the proliferative index pattern of Ki-67, which is routinely used to aid diagnosis in morphologically difficult cases, for example between LSIL and reactive or metaplastic lesions (32). In certain cases, we found similar expression patterns of ADAR1 and Ki-67, with basal and parabasal layer positivity in LSILs, which indicated that ADAR1 positivity was related to an increase in proliferative index, although it was limited to the lower epithelial layers (Figure 2B). ADAR1 expression further increased during disease progression (Figure 2A, panels e–j) and extended to the upper layer of squamous epithelium, and Ki-67 showed a similar distribution pattern, alongside p16, a surrogate marker of HPV infection and high-grade dysplasia (data not shown).

We also investigated whether the increasing expression of ADAR1 was accompanied by changes in its staining pattern, since ADAR1 is localized in the nucleus and/or the cytoplasm. We observed that in HSIL/in situ carcinomas (HSIL/CISs) and in ICs, ADAR1 showed cytoplasmic positivity significantly different from that in LSILs (Figure 2C). Moreover, there was a decrease in nuclear positivity, particularly between LSIL and CIS. Within the same group of lesions, the increase in cytoplasmic versus nuclear expression was statistically significant in CIS and IC, but not in LSIL (Figure 2C, *P* = 0.004 in CIS; *P* = 0.017 in IC). ADAR1 expression was even more evident in high-grade (grade 3 [G3]) compared with G2 CC (Figure 2D), although our consideration was semiquantitative.

Considering our interest in tumor-infiltrating innate lymphocytes, in more aggressive tumors we also examined the presence of cells expressing CD56, a well-known NK cell marker. Separating tumors on the basis of a low (CD56^+^ <5) or not low (CD56^+^ >5) number of positive cells, we observed a significantly higher percentage of ADAR1^+^ cells in G3 tumors in the group with low CD56^+^ cells (Figure 2E). Together, these results showed increasing expression of ADAR1 during disease progression from LSIL to HSIL/CIS and IC. Moreover, in samples from the latter group of patients, ADAR1 expression was even higher in G3 compared with G2 tumors, with a significant difference also maintained when lesions with low CD56^+^ cells were analyzed.

### Decreased levels of tumor-infiltrating ILCs are observed in CC.

**Figure 3:**
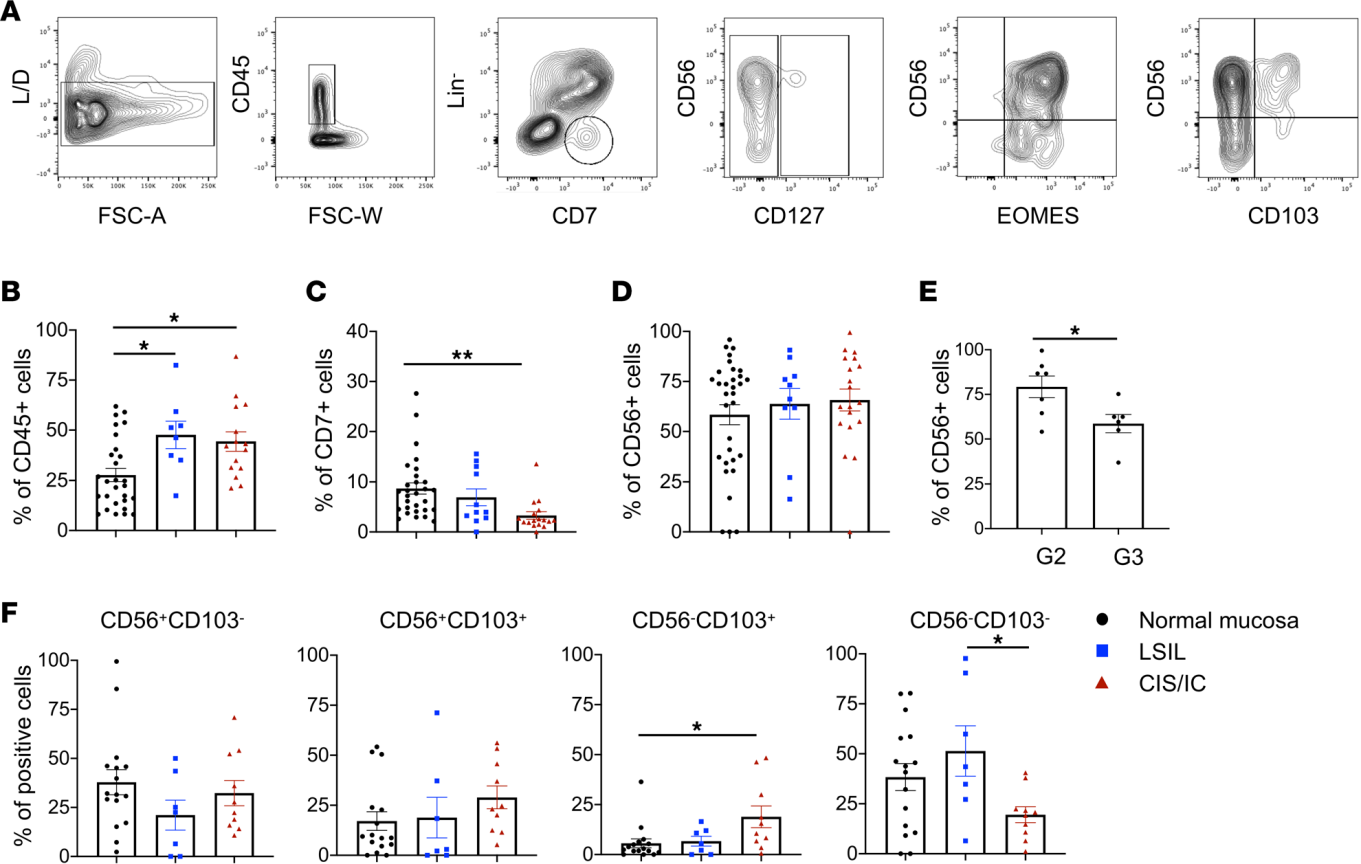
Analysis of innate immune cells infiltrating LSIL, HSIL/CIS, and IC. Fresh biopsy samples obtained from different groups of patients were analyzed for the presence of an innate immune cell infiltrate. (**A**) Gating strategy from representative FACS plots, showing the isolation of (left to right panel) live cells, CD45^+^ cells (gate excludes monocytes), and Lin^–^CD7^+^ ILCs (middle right panel), further divided according to CD56, CD127, EOMES, and CD103 expression. ILCs were thus defined as Lin^–^CD45^+^CD7^+^ and then separated into NK/ILC1, ILC2, and ILC3 subpopulations (see the main text for more details). (**B** and **C**) Bar graphs represent the percentage of CD45^+^ (**B**) and CD7^+^ (**C**) cells among each group of patients. HSIL/CIS and IC biopsy samples were grouped together (CIS/IC, red triangles) and compared with LSIL or normal mucosa used as control. (**D** and **E**) Bar graphs represent the percentage of CD56^+^ cells among the Lin^–^CD45^+^CD7^+^cells in the different groups of patients (**D**) and in G2/G3 tumors (**E**). (**F**) Bar graphs represent the percentage of distinct subsets expressing or not CD56 and CD103. Histograms represent mean ± SEM. *P < 0.05, **P < 0.01. Two-way ANOVA was used for multiple comparisons. Each symbol represents a single biopsy sample.

To further investigate immune cells infiltrating cervical lesions, we analyzed the proportion of ILCs isolated from fresh biopsy samples obtained from different groups of patients (Figure 3). In situ and invasive carcinomas were grouped together and compared with LSILs, as well as with normal mucosa used as control. Total leukocytes were identified as CD45^+^ cells, and within them innate lymphocytes were gated as CD7^+^ and negative for T cell (CD3, CD4, CD5), B cell (CD19), and monocyte (CD14) markers. Although an increased percentage of infiltrating CD45^+^ cells characterized both LSIL (44%) and CIS/IC (37%) compared with normal mucosa (32%), the frequency of innate CD7^+^ lymphocytes was significantly decreased in CIS/IC (from ~8% in normal mucosa, to ~7% in LSIL, and to ~3% in CIS/IC) (Figure 3, B and C). We further characterized CD7^+^ cells to discriminate between NK, ILC1, ILC2, and ILC3 cell populations. In humans, the distinction between NK and ILC1 cells can be challenging, but it is widely accepted that tissue ILC1, ILC2, and ILC3 cells can be identified by the expression of IL-7Ra (CD127) in combination with group-specific TFs: EOMES, GATA3, and RORγt, respectively. Analysis of CD7^+^ infiltrating lymphocytes showed a very low percentage (0%–5%) of CD127^+^ cells regardless of the biopsy sample analyzed and undetectable GATA3 and RORγt expression, thus ruling out the presence of ILC2 and ILC3 populations. On the other hand, approximately 60% of CD7^+^ cells coexpressed the NK marker CD56 and the NK TF EOMES, leading to their identification as NK cells, although no marked differences were detected between control mucosa, LSIL, and CIS/IC samples (Figure 3, A and D, and data not shown). However, we noticed a significantly lower percentage when more aggressive (G3) ICs were analyzed (~60% in G3 versus ~80% in G2) (Figure 3E).

NK cells can recirculate from blood to tissues, where they can be quickly recruited during viral infection or tumor growth, and, as with other lymphocytes, their tissue retention is facilitated by CD69, CD103 (αE integrin), and CD49a expression. Therefore, to investigate the possible tissue-resident nature of infiltrating NK cells, we further analyzed CD7^+^ cells for expression of CD103 in combination with CD56 (Figure 3F). There was a general rise in CD103^+^ cells in CIS/IC biopsy samples, with a statistically significant difference reached in the CD56^–^ subset (with a 3-fold increase compared with normal mucosa or LSIL). This increase was accompanied by a statistically significant decrease in the CD103^–^CD56^–^ subset (from ~55% in LSIL to ~20% in CIS/IC). These cells were, however, NK cells, as they maintained expression of the typical NK markers EOMES and CD94.

**Figure 4:**
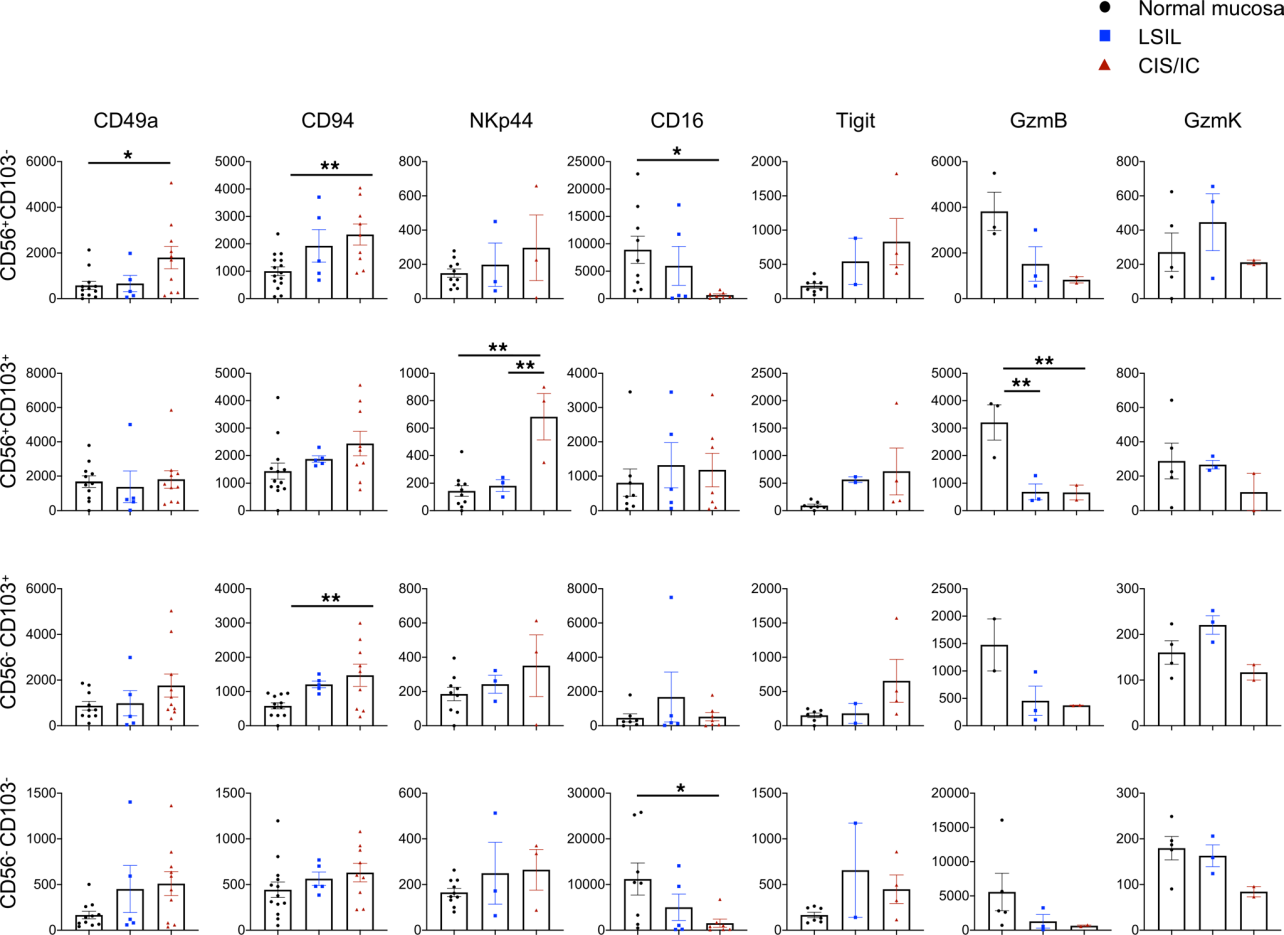
Phenotypic characterization of ILC1/NK cell subsets during CC progression. MFI of selected marker expression by distinct subsets (CD56^+^CD103^–^, CD56^+^CD103^+^, CD56^–^CD103^+^, and CD56^–^CD103^–^). Histograms represent mean ± SEM. *P < 0.05, **P < 0.01. Two-way ANOVA was used for multiple comparisons. Each symbol represents a single biopsy sample.

We further broadened the phenotypic characterization of the CD56/CD103 subset and assessed expression of some activating/inhibitory receptors (CD16, NKG2D, NKp46, NKp44, NKp30, KIR, CD94, Tigit) and adhesion (CD49a) and cytotoxic molecules (GzmB and GzmK). The major modulations were observed in the CD56^+^ cell subsets, where an increase in the expression of CD49a, CD94 and NKp44 alongside a decrease in the activating receptor CD16 and in GzmB expression characterized CIS/IC-infiltrating cells (Figure 4).

Collectively, these data suggest that the innate CD7^+^ lymphocytes infiltrating the uterine cervix were NK cells, and their frequency appears to have been reduced in more aggressive G3 tumors. They also highlight the role of the TME in shaping their phenotype by promoting the acquisition of tissue-resident features (CD103, CD49) and/or by impacting effector functions through alterations of the cells’ activating receptor expression profile and cytotoxic molecule content.

### ADAR1 silencing in CC-derived cell lines induces the expression of IFN-stimulated genes and affects cell proliferation.

As our results demonstrated not only increased ADAR1 expression during CC progression but also a reduction in NK cell infiltration in tumors, we aimed to establish an in vitro system to better investigate the impact of ADAR1 expression on innate lymphocytes, particularly NK cells. Data from the Cancer Cell Line Encyclopedia (CCLE) allowed us to select CC-derived cells suitable for our experiments. Based on expression pattern and *z* score, indicating the gene’s expression level compared with the mean in each cell line, we selected SiHa and CaSki cells among those with higher and lower levels of *ADAR1* expression, respectively (Supplemental Figure 1, A and B).

Next we sought to determine whether SiHa and CaSki cells exhibited ADAR1 dependency in terms of survival by analyzing available CRISPR/Cas9 datasets (24), and according to The Cancer Dependency Map (DepMap), *ADAR1* appeared to have an essential function in both cell lines (Chronos score < –0.5) (Supplemental Figure 1C).

To investigate the effects of ADAR1 manipulation on CC-derived cells and on innate immune cells and pathways, we set up an in vitro system where ADAR1 expression was transiently inhibited by specific siRNA (siADAR1). ADAR1 silencing in both SiHa and CaSki cell lines showed a knockdown efficiency of approximately 50%–70%, as demonstrated by RT-PCR and immunoblot analysis. As expected, a consequence of ADAR1 depletion was induction of a type I IFN response, evidenced both by an increase in *IFNβ* transcript levels and by PKR phosphorylation at Thr446/Thr451 residues (Supplemental Figure 2, A and B).

**Figure 5:**
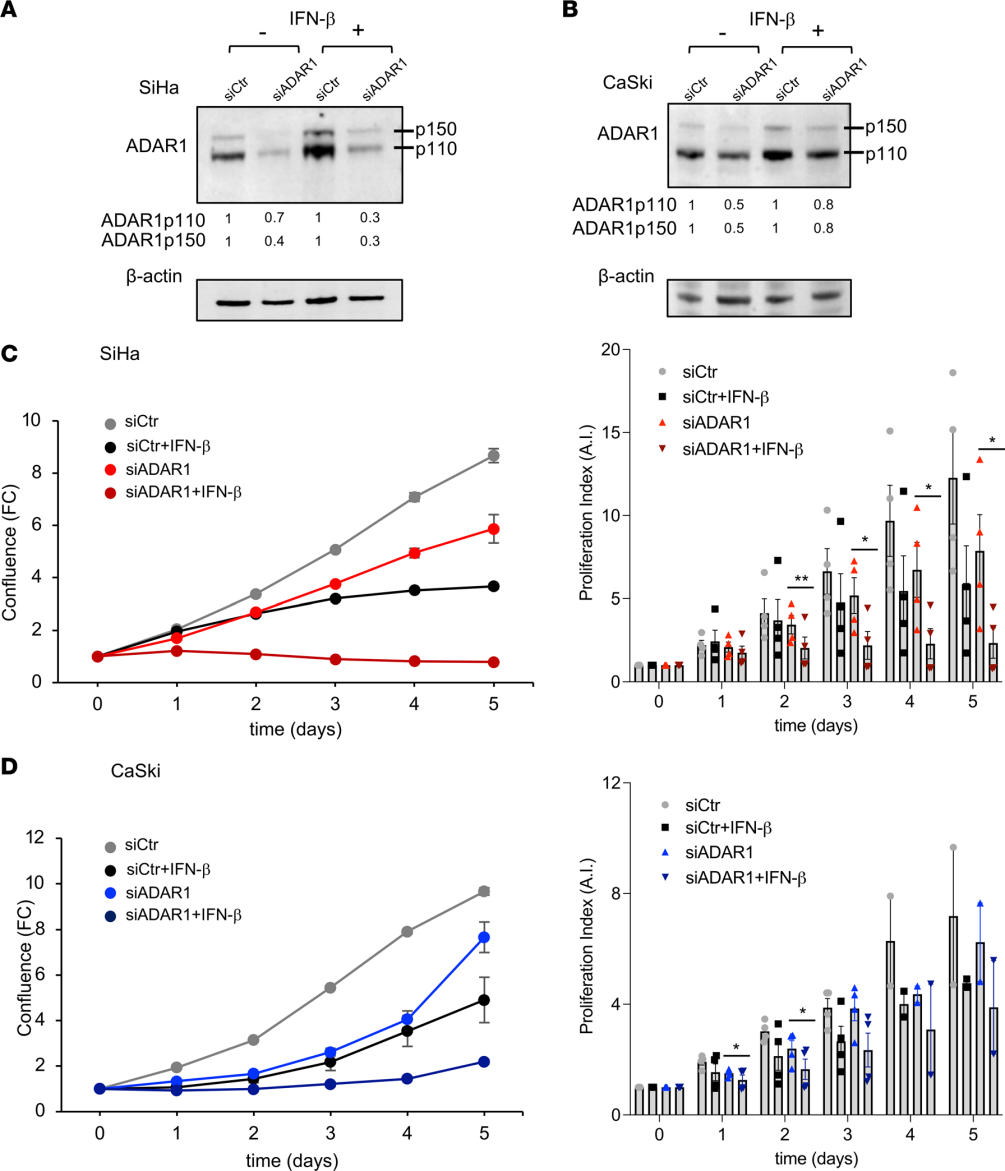
Effects of IFN-β on CC-derived cell proliferation upon *ADAR1* silencing. SiHa and CaSki cell lines were transfected with an ADAR1 siRNA for 72 hours (considered as day 0), then treated with IFN-β (1,000 IU/mL) for several days. ADAR1 expression was analyzed by immunoblotting after 48 hours on SiHa (**A**) or CaSki (**B**) cells. (**C** and **D**) Proliferation was monitored up to 5 days by Incucyte Live-Cell Analysis, analyzed for cell confluence, and expressed as fold change (FC) normalized to the scan time 0 (T0) set as 1, using Incucyte Zoom software. Proliferation index was calculated by setting cells at T0 as 1 (arbitrary index [A.I.]). Results from one representative experiment and pooled data from 4 independent experiments (mean ± SEM) are shown. *P < 0.05, **P < 0.01.

We then investigated the ADAR1 dependency of SiHa and CaSki cells by long-term proliferation assays using the Incucyte imaging system, which showed a decrease in proliferative activity of ADAR1-silenced cells in both cell lines (Figure 5, C and D). As loss of ADAR1 can cause both cell-intrinsic lethality (e.g., via high levels of ISGs, including PKR) and induction of key antitumor cytokines, including IFN-I, we asked whether administration of IFN-I in the context of ADAR1 loss would further limit cell proliferation (24). Both cell lines were treated with IFN-β, starting 3 days after siRNAs transfection (day 0) and up to 5 days (Figure 5, A and B), and cell proliferation was then measured. Indeed, in the presence of IFN-β, both ADAR1-silenced cell lines were sensitive to the treatment, as their proliferative capacity was even further inhibited compared with that of all other combinations (Figure 5, C and D).

To further explore other relevant changes in gene expression profiles caused by ADAR1 silencing, we performed an RNA-Seq analysis (Supplemental Figure 3). Inhibition of ADAR1 resulted in increased expression of 1,410 and 829 genes in SiHa and CaSki cells, respectively, while 1,540 and 346 genes were downmodulated (logFC > 1; FDR < 0.05) (Supplemental Figure 3, A and B). Among the induced genes, we could identify numerous *ISGs*, including several proinflammatory cytokines and chemokines involved in the regulation of innate lymphocytes, including NK cells (e.g*.*, *CXCL8*, *CXCL9/10/11*, *CCL5*, *IL12*, *IL18*, *IL6*) (Supplemental Figure 3, C and D). KEGG pathway enrichment analysis showed similar functional enrichment signatures, confirming the activation of several pathways related to the inflammatory response, such as cytokine–cytokine receptor interaction, *NF-kB* signaling pathway, and *TNF* signaling pathway (Supplemental Figure 3, E and F).

Overall, these data suggest that ADAR1 inhibition in CC cells can cause both a constraint of their cell-proliferative capacity and upregulation of proinflammatory cytokines and chemokines associated with innate immunity and antitumor effects.

### Conditioned medium from ADAR1-silenced CC-derived cell lines enhances NK cell effector functions.

**Figure 6:**
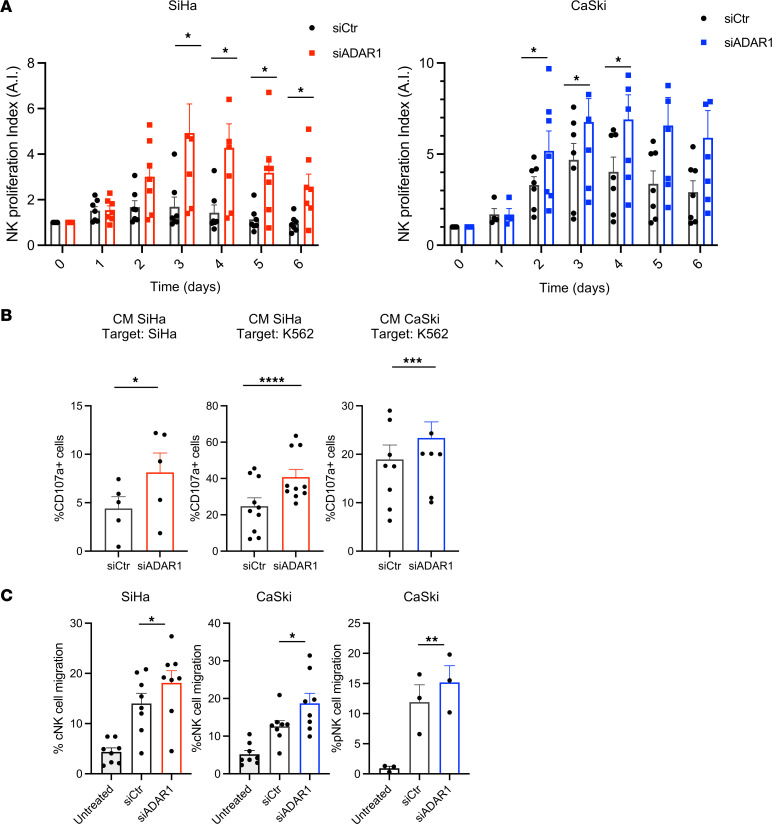
ADAR1 inhibition enhances NK cell functions. Conditioned media (CM) from siCtr^–^ and siADAR1-transfected SiHa and CaSki cells were harvested after 72 hours and used in different assays. (**A**) CM from SiHa and CaSki cells was incubated for the indicated time points with purified NK cells isolated from healthy donors. Proliferation was measured by Incucyte Live-Cell Analysis and analyzed with Incucyte Zoom software. Proliferation index was calculated as fold change by setting Nuclight-positive NK cells at scan 0 (T0) as 1 (A.I.). Pooled data are from 3 independent experiments with NK cells from 7 different donors. (**B**) NK cell degranulation was evaluated by FACS using the lysosomal marker CD107a. Purified NK cells were used as effectors and treated with CM from SiHa or CaSki cells for 18 hours and then cocultured with SiHa (left panel) or K562 (middle and right panels) cells, used as targets (E/T ratio of 1:2). CD107a expression was evaluated on NK cells gated as CD56^+^. Pooled data are from 3 independent experiments with NK cells from 6 (target SiHa) or 7 (target K562) different donors. (**C**) Migration of cultured (cNK) or primary (pNK) purified NK cells was measured using a Transwell migration chamber. As chemoattractant, SiHa and CaSki CM was added to the lower compartment, and after 2–4 hours at 37°C, the migrated cells were counted using BD FACSCanto. Pooled data are from at least 2 independent experiments with 7 different donors. All data are expressed as mean ± SEM. Statistical analysis was performed by paired *t* test. *P < 0.05, **P < 0.01, ***P < 0.001, ****P < 0.0001.

In view of the induction of *IFN-I*, *ISGs*, and proinflammatory factors observed in the transcriptomic analysis of ADAR1-silenced cells, we asked whether conditioned media (CM) collected from these cells could influence NK cell effector functions. Thus, CM from siADAR1 or siCtr SiHa and CaSki cells were collected at 72–96 hours after transfection and then used to stimulate NK cells. First, freshly purified NK cells were incubated with CM, and their proliferative capacity was analyzed for several days by the Incucyte imaging system. The results showed a significantly higher rate of NK cell proliferation in the presence of siADAR1- compared with siCtr-derived cell supernatants (Figure 6A).

Then, we determined the effects of CM on NK cell–mediated killing. Freshly isolated and purified NK cells were cultured overnight with different CM and then used as effector cells in degranulation assays. As targets, we used either the same CC-derived cell line from which the CM was collected or K562 cells, the prototypic target of human NK cell–mediated killing. As an indicator of NK cell degranulation, the expression of the widely used marker CD107a was evaluated by FACS analysis gating on viable CD56^+^ NK cells after 4 hours of incubation with target cells. As shown in Figure 6B, NK cells stimulated with siADAR1 CM derived from SiHa cells showed a higher level of degranulation against SiHa or K562 cells compared with NK cells incubated with siCtr CM. Similarly, siADAR1 CM derived from CaSki cells activated NK cell degranulation against K562, while CaSki cells were resistant, as induction of CD107a expression was always below 4% (data not shown). In a parallel set of experiments, we addressed the role of IFN-I possibly released in the supernatants of siADAR1 cells in the activation of NK cell degranulation. NK cells were pretreated with a blocking anti-IFNAR2 mAb or an isotype control IgG, then incubated with CM derived from SiHa cells for 18 hours and tested against K562 target cells. Our findings indicate that blocking the IFN-I receptor on NK cells resulted in statistically significant inhibition of the degranulation triggered by siADAR1 CM (*P* = 0.0014 between siADAR1/IgG and siADAR1/anti-IFNAR2), while it had no effect with siCtr CM (Supplemental Figure 4A). Moreover, the effects of siCtr CM were similar to those observed on NK cells cultured with fresh medium. As a control of the blocking capacity of the anti-IFNAR2 mAb, we also tested the degranulation activity of NK cells previously stimulated with IFN-β (100 IU/mL, for 18 hours) against K562 targets (Supplemental Figure 4B). As expected, IFN-β increased CD107a expression on NK cells, which was significantly inhibited by incubation with the blocking mAb (*P* = 0.0018 between IgG and anti-IFNAR2), returning to the basal levels observed when NK cells were cultured with fresh medium alone. No CD107a expression was detected when NK cells were cultured without target cells, independently of IFN-β stimulation. Finally, we also analyzed the effect of CM on NK cell migration. Primary or cultured NK cells incubated with supernatants from siADAR1 SiHa and CaSki cells showed an increased ability to migrate compared with siCtr medium–incubated cells (Figure 6C).

Together, the results obtained with CM from CC-derived cell lines suggest that inhibition of ADAR1 expression induced the release of IFN-I and of other soluble factors able to boost NK cell proliferation, killing, and migration.

### ADAR1 inhibits NK cell infiltration and cytotoxic capacity in 3D organotypic cultures.

**Figure 7:**
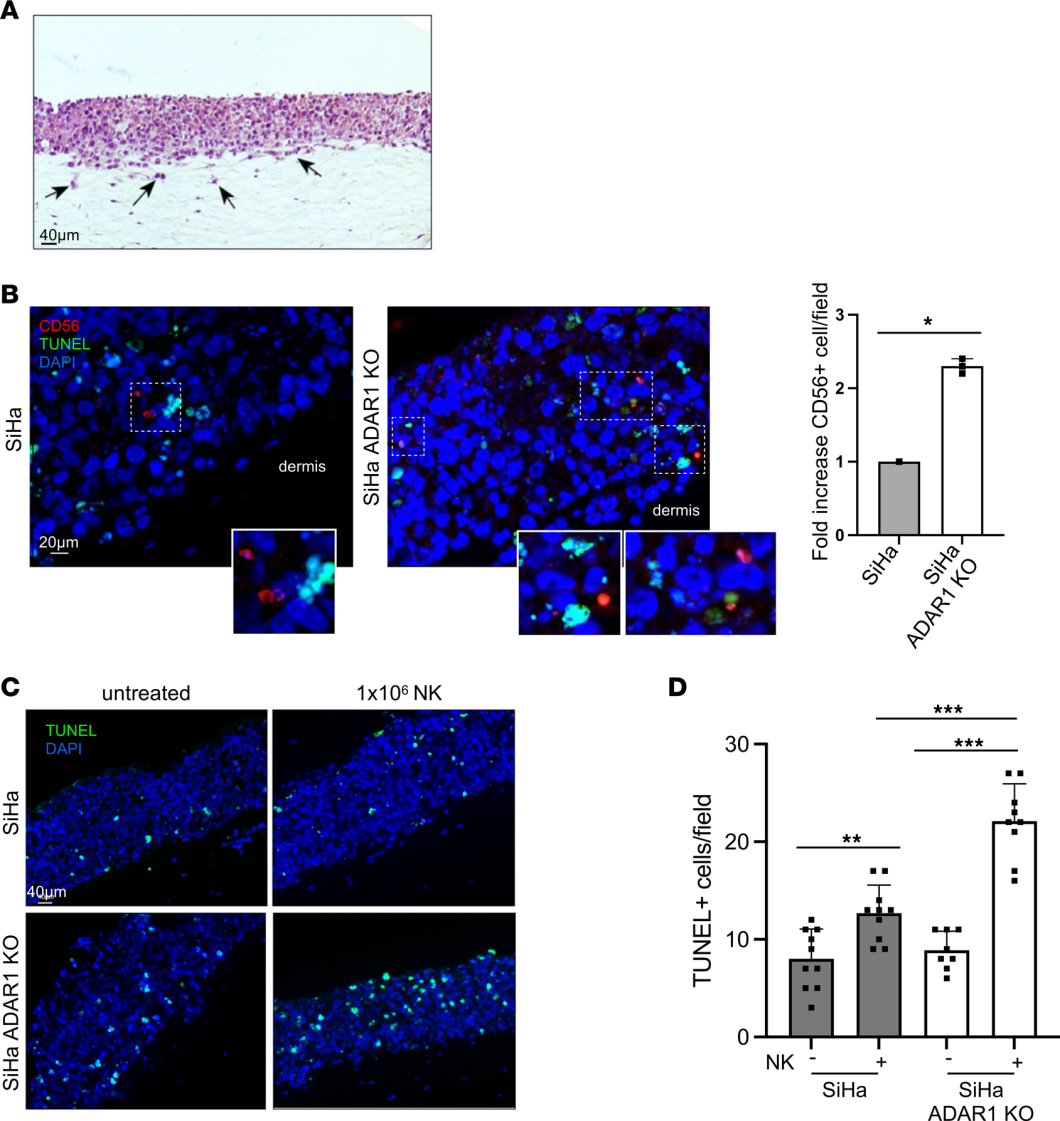
Impact of ADAR1 silencing and of NK cell infiltration on SiHa cell apoptosis in organotypic cultures. 3D cultures of ectocervical squamous epithelia equivalents were prepared using control SiHa and SiHa *ADAR1*-KO cells (KO by CRISPR/Cas9). After 3 weeks, NK cells were added on the top of stratified layers and left to infiltrate for 24 hours (see Methods for details). Rafts were finally fixed, embedded in paraffin, and stained with H&E or processed for immunofluorescence and TUNEL assays. (**A**) Representative image of a SiHa raft section stained with H&E, showing the highly irregular basal layer of the epithelial portion and invasive events in the matrix counterpart (arrows). Scale bar: 40 μm. (**B**) Sections were stained using anti-CD56 antibody (red) and TUNEL (green). Nuclei were counterstained with DAPI. Bar graphs show the fold increase in infiltrating CD56^+^ cells/field (mean ± SEM). Scale bar: 20 μm. (**C** and **D**) Sections were also quantified for TUNEL^+^ cells (green) on low-magnification images of each sample. Bar graphs show the number of TUNEL^+^ cells/field ± SD. Scale bar: 40 μm. Statistical analysis was performed by paired *t* test. *P < 0.05, **P < 0.01, ***P < 0.001.

To further investigate the impact of ADAR1 expression on NK cell effector functions, we set up 3D organotypic cultures and tested the ability of NK cells to infiltrate the epithelial layers and induce cell apoptosis. Ectocervical epithelium equivalents that could efficiently mimic HSIL in vitro were prepared using *ADAR1*-KO or control SiHa cells (Supplemental Figure 5). In Figure 7A, a representative image of SiHa raft sections stained with H&E shows the highly irregular profile of the epithelial basal layer, with several events of matrix invasion, confirming the high invasive potential of SiHa cells. Next, we analyzed the ability of NK cells to infiltrate the in vitro generated epithelium expressing or not expressing ADAR1. To this aim, we initially prepared organotypic raft cultures using SiHa cells knocked out or not for *ADAR1*, and after 21 days, we added NK cells on the top of the cultures. Twenty-four hours after NK cell seeding, we performed quantitative immunofluorescence analyses using an anti-CD56 antibody to visualize infiltrating NK cells and TUNEL assay to identify apoptotic nuclei. The data revealed that in *ADAR1*-KO cultures, the number of CD56^+^ cells detected in the rafts was slightly but significantly higher than in control samples (Figure 7B), and they appeared to be surrounded by a greater number of TUNEL-positive nuclei and nuclear fragments (Figure 7B, insets). Quantitative analysis of TUNEL staining, performed using lower-magnification images for each sample, confirmed that *ADAR1*-KO cultures displayed a significant increase in apoptotic nuclei in the presence of NK cells, compared with controls (Figure 7, C and D). Overall, these results suggest that in 3D organotypic cultures, ADAR1 depletion enhanced the ability of CC-derived cells to be recognized and targeted by activated NK cells.

## Discussion

In the present study, to investigate the mechanisms involved in the suppression of innate immune responses that may favor tumorigenesis, we explored, for the first time to our knowledge, the interplay among ADAR1, IFN-I, and NK cells. We focused on CC, since its progression from the SIL premalignant condition to in situ and then invasive carcinoma may span 10 to 20 years after HPV infection, thus reflecting a long-lasting inability of immune responses to eliminate infected cells and highlighting at the same time an ample window for potential therapeutic interventions. In this context, ADAR1 may play a central role by its ability to dampen IFN-I responses, which have well-known antitumor effects (16). In fact, ADAR1 overexpression is well documented in several cancers, and it is correlated with clinically aggressive behavior and poor prognosis (21). Regarding CC, despite the limited number of studies published to date, a similar trend appears to emerge, suggesting that patients with high ADAR1 expression have a worse prognosis (28, 29). This scenario may be linked to the suppressive effects mediated by ADAR1 on the TME, as a previous study in other models demonstrated that loss of ADAR1 enhances tumor inflammation and immune cell infiltration (19, 26).

Investigating publicly available datasets, we indeed observed an inverse correlation between *ADAR1* expression and patients’ overall survival probability, along with a progressive decrease in expression of a set of *ISGs* from stage I to stage IV tumors. These preliminary observations were further addressed in a panel of cervical lesions of different types and grades, where ADAR1 expression, while almost undetectable in normal mucosa, was highly enhanced in the progression from LSIL to IC and further increased in more aggressive, G3 tumors. We also noticed an alteration of its cellular distribution during SIL to IC progression, with a significant increase in the percentage of cytoplasmic ADAR1^+^ cells. In this regard, it is well known that *ADAR1* undergoes alternative splicing, resulting in a constitutively expressed p110 short isoform primarily localized in the nucleus, and in a long IFN-inducible p150 isoform that can shuttle between the nucleus and the cytoplasm, where it predominantly resides (20, 22). However, it remains to be clarified whether the variation in ADAR1 cellular distribution correlates with expression of a specific isoform, IFN-I levels in the TME, changes in the editing activity, and/or patient prognosis. Nevertheless, the immunoregulatory role of ADAR1 in the progression of CC is supported by additional observations. Specifically, when analyzing ADAR1 expression in G2 and G3 tumors with fewer infiltrating CD56^+^ cells (CD56^+^ < 5), we observed significantly higher ADAR1 expression in the more aggressive G3 lesions. Moreover, leukocytes isolated from fresh biopsies exhibited a progressive reduction in the percentage of infiltrating innate lymphocytes, from normal mucosa, to LSIL, and finally to tumors. These cells were identified as NK cells based on their expression profile (i.e., Lin^–^CD45^+^CD7^+^EOMES^+^CD56^+/–^), and a subset of them expressed CD103 and CD49a tissue-resident markers and were further characterized by higher expression of CD94 and NKp44, as well as of the inhibitory receptor Tigit. Additionally, there was a reduction in CD16 and GzmB expression when compared with cells isolated from normal mucosa. Collectively, these findings suggest that NK cells infiltrating tumors strengthen their tissue-resident features, downregulate their main activating receptor and cytotoxic molecules, and increase Tigit inhibitory receptor, thus indicating an alteration of NK cell functionality. Furthermore, the significant reduction in the percentage of freshly isolated CD56^+^ cells in G3 compared with G2 tumors, accompanied by our previous observations on tumor-infiltrating CD56^+^ cells in IHC analysis, may represent a feature of CC progression. Indeed, more recent single-cell transcriptome studies investigating the composition of TME during CC progression demonstrated that the impaired local immune landscape plays a key role in cervical carcinogenesis. Moreover, although to our knowledge, no data have been reported on other ILC populations, NK cells displayed notable stage-dependent differences in tumors. In general, HSILs were characterized by an activated TME, infiltrating effector NK cells, and a proinflammatory signature, while ICs showed an immunosuppressive TME with resident NK cells (33). Of note, analysis of tumors and paired adjacent stromal tissues revealed a slight enrichment of the NK cell subset with a suppressive phenotype in the tumor area, while the subset with an activated cytotoxic phenotype was excluded (13). TME composition in CC may also be influenced by treatment strategies, as single-cell RNA-Seq of CC biopsies before and after radiochemotherapy revealed an increase in *CD16*^+^ NK cells, which exhibited an enhanced cytotoxic (*GRZB/H*) and migration (*CCL5/CCR1*) gene signature following therapy (12). Integrative spatial transcriptomics and proteomics analysis uncovered bidirectional tumor stroma–immune system interactions, where malignant cells interacted with NK/T cells through IFN signaling, and revealed extensive cellular communications — based on chemokine/chemokine receptors — between tumor and immune cells, including NK cells (14). Indeed, preliminary analysis of samples from a CC clinical trial (NCT04516616) demonstrated that neoadjuvant chemotherapy (NACT) induced a state transition characterized by increased tumor-infiltrating immune cells, which correlates with response to immune-checkpoint blockade, at least partly through IFN activation. Therefore, some of the tumor states identified not only were correlated with immune cell infiltration abundance but might also be directly involved in recruiting immune cells. The results of our ex vivo analysis indicate a decrease in NK cells in more aggressive tumors and highlight a potential role for ADAR1 in modulating the TME. In fact, transcriptomic analysis on CC-derived cell lines revealed that *ADAR1* silencing pushed cancer cells toward a proinflammatory phenotype through the induction of *IFN-I*, *ISGs*, and lymphocyte-recruiting chemokines and cytokines known to be involved in NK cell recruitment and activation (34). Accordingly, Zhang et al. performed an unsupervised hierarchical clustering of various immune cell types in CC and identified a unique subset of patients with disproportionate intratumoral NK cells and a significantly lower risk of tumor progression (10).

We reasoned that this inflamed microenvironment triggered by ADAR1 silencing could influence activation of NK cell functions, an aspect that to our knowledge has not been addressed in any tumor model. Indeed, exposing NK cells to CM harvested from ADAR1-silenced cells significantly enhanced their proliferation, killing, and migration. Although the role of specific cytokines/chemokines in regulating NK cell functions was not investigated, we believe the cooperative action of several factors, likely including IFN-I, may be required. Type I IFNs play central roles in the immune system’s defense against tumors, and their antitumor effects are multifaceted, involving both indirect and direct mechanisms such as activation/attraction of infiltrating immune cells and induction of apoptosis and of cell cycle arrest in tumor cells (16). Indeed, the inhibition of SiHa and CaSki cell proliferation, already detectable upon ADAR1 silencing, was even more marked when we treated silenced cells with IFN-β. This suggests that ADAR1 inhibitors could synergize with existing anticancer immunotherapies based on IFNs and on other proinflammatory factors to arrest cancer progression and potentiate antitumor NK cell immune responses. In this context, a recent study on the B16 murine model demonstrated that ADAR1 suppression in cancer cells had a profound impact on TME and enhanced antitumor immunity, as it caused a global reshaping of tumor-infiltrating lymphocytes and sensitized tumors to immunotherapy and to IFNs (19). In our model of 3D organotypic cultures, we also detected a significant increase in apoptotic nuclei in the context of *ADAR1* KO and upon infiltration of NK cells. Thus, the possibility to boost NK cell recruitment and activation in tumors via ADAR1 downregulation may allow development of alternative antiviral/anticancer therapies.

Regarding the initiation and progression of tumors in the cervix, while the role of the immune system in controlling HPV is well established, recent studies highlight a more complex scenario. These studies suggest that chronic inflammatory responses initiated by HPV-transformed cells can reprogram the local immune environment, thereby fueling cancer progression (35). In fact, precancerous high-grade lesions, as well as invasive CCs, are frequently associated with strong inflammatory infiltrates in the stroma. This inflammatory milieu, while initially part of the body’s defense mechanism, can paradoxically promote tumor progression at later stages. Thus, the immune system emerges as a double-edged sword also in HPV-associated carcinogenesis, with its role changing in a stage-dependent manner. ADAR1 appears to be well adapted to this scenario, and we propose that — within the TME — a fine-tuning exists between chronic inflammation and production of inflammatory factors that sustain ADAR1 expression on one hand, and inhibition of cytokine release by ADAR1 itself on the other. Thus, our data suggest that induction of an acute — more than a chronic — inflammatory TME stimulated by ADAR1 inhibition might contribute to cancer cell death by inducing both cell cycle arrest and antitumor immune responses.

In summary, these findings reveal a previously unrecognized role of ADAR1 as a potential therapeutic target affecting HPV^+^ CC cells. Additionally, they provide proof of concept that silencing ADAR1 can be combined with standard chemotherapies, as well as with IFN and proinflammatory therapies, to enhance their antiproliferative and anticancer effects, potentially extending beyond CC.

## Methods

Further information is provided in Supplemental Methods.

### Sex as a biological variable.

Our study exclusively examined samples from female donors, because CC is a disease that affects women.

### Enrollment of patients and IHC stainings.

For the retrospective study, we selected from our database 68 patients referred to the Department of Gynecological, Obstetrical, and Urological Sciences of the Umberto I Hospital in Rome between the years 2017 and 2019. Patients had a diagnosis of LSIL (*n* = 17), HSIL/CIS (*n* = 10), or IC (*n* = 41, of which 34 were squamous carcinomas and 7 adenocarcinomas); the latter patients underwent 3 cycles of platinum-based NACT (weekly 30 mg/mq cisplatin plus 60 mg/mq paclitaxel), followed by radical hysterectomy (Supplemental Table 2). Clinical information, comprising age, clinical stage, and pathologic response to neoadjuvant treatment, including pathological staging (36) and grading definition (*AJCC Cancer Staging Manual*, 8th ed., 2017) were obtained from the institutional databases. Two experienced pathologist reviewed histological features. For diagnostic purposes, the diagnosis of LSIL was confirmed by Ki-67 expression on basal and parabasal layers of squamous epithelium, while HSIL showed diffuse expression of Ki-67 and overexpression of p16. Overexpression of p16 in infiltrating carcinomas was considered a surrogate marker of HPV association (37, 38).

Serial sections were obtained from each paraffin block for IHC evaluation of ADAR1 expression and CD56^+^ infiltrating cells. Hematoxylin was used for cytoplasmic and nuclear counterstaining. ADAR1 immunostainings were performed with mouse mAb sc-271854 (dilution 1:100, Santa Cruz Biotechnology), using an automated immunostainer (BOND-MAX, Leica Microsystems) with the BOND Polymer Refine Detection kit according to the manufacturer’s instructions. Negative controls were obtained by omitting the primary antibody. A minimum of 200 neoplastic cells were present in each biopsy sample. A positive stain was defined as the presence of nuclear and/or cytoplasmic staining, either strong or weak, complete or incomplete, in at least 1% cells. For each case, the staining of the entire section was semiquantitatively assessed using the H-score method as follows: H-score = (3 × percentage of tumor cells with 3^+^ staining) + (2 × percentage of tumor cells with 2^+^ staining) + (1 × percentage of tumor cells with 1^+^ staining), in the nucleus and/or in the cytoplasm of the entire section. This score, therefore, is in the range of 0 to 300. Evaluation of infiltrating CD56^+^ cells was carried out with the anti-CD56 antibody (PA0191) (Bond RTU Primary, Leica Microsystems). All tests were evaluated independently by A. Pernazza and M. Leopizzi.

### Characterization of infiltrating leukocytes in fresh biopsies.

Fresh biopsy samples from LSIL (*n* = 14), HSIL/CIS (*n* = 10), IC (*n* = 18), or normal mucosa (*n* = 40) were obtained from women enrolled at the Department of Gynecological, Obstetrical, and Urological Sciences of the Umberto I Hospital in Rome (Supplemental Table 3), with the following inclusion criteria: no systemic diseases, no immunodeficiency, being fertile and sexually active with no current pregnancy, intact uterus, no use of antibiotics or vaginal antimicrobials in the previous month, no vaginal intercourse or vaginal lavage within the last 3 days, and no treatment for cervical disease or other sexually transmitted infections in the previous 6 months. Samples were obtained during surgical treatment and immediately treated for leukocyte isolation. Tissue samples were washed in PBS, minced with a scalpel, treated with Tumor Dissociation Kit solution, and processed with gentleMACS Octo Dissociator (all from Miltenyi Biotec) for 1 hour at 37°C. Cells were filtered through a 100 μm cell strainer filter and treated with red blood cell lysis buffer for 10 minutes at room temperature. Cells were then counted and stained for FACS analysis. Samples containing fewer than 1,000 CD45^+^ cells were excluded from analysis due to low event count.

### FACS analysis.

Cells freshly isolated from biopsy samples were stained with antibodies specific for extracellular antigens and resuspended in Brilliant Stain Buffer (BD Horizon) for 30 minutes at 4°C. They were then fixed and permeabilized with FOXP3 staining buffer for 20 minutes at room temperature and finally stained with antibodies specific for intracellular antigens resuspended in the FOXP3 washing buffer for 30 minutes at 4°C (eBioscience FOXP3/Transcription Factor Staining Buffer Set, Invitrogen). Fixable viability dye (BD Biosciences) was used to discriminate between live and dead cells; negative cells were considered viable. Innate lymphocytes were identified among lineage negative (Lin^–^) (CD3^–^CD14^–^CD19^–^CD5^–^CD4^–^) CD45^+^CD7^+^ cells. Type 1 ILCs were identified according to CD56, CD127, EOMES, and T-BET expression. Sample acquisition was performed by a LRSFortessa flow cytometer (BD Biosciences), and data were analyzed with FlowJo software. A detailed list of mAbs used is provided in Supplemental Table 4.

### Cell cultures.

SiHa, CaSki, and K562 cell lines and primary cultures of human fibroblasts (HFs) are described in Supplemental Methods. Human NK cell cultures were obtained by coculturing PBMCs with irradiated RPMI8866 feeder cells for 8–10 days (39). Primary human NK cells were purified from PBMCs by negative selection using the RosetteSep Human NK Cell Enrichment Cocktail (STEMCELL Technologies). Proliferation, migration, and cytotoxicity experiments were performed with NK cell populations that were 80%–95% CD56^+^CD3^–^.

### siRNA and ADAR1 KO.

CaSki (2 × 10^5^) and SiHa (3 × 10^5^) cells were transfected using Oligofectamine Transfection Reagent (Thermo Fisher Scientific) charged with a mixture of 3*ADAR1*-specific siRNAs (siADAR1) (sc-37657) or a nontargeting siRNA (siCtr) (sc-37007, both from Santa Cruz Biotechnology) (30 nM final concentration). *ADAR1* gene expression levels were evaluated at 72–96 hours after transfection by RT-PCR or immunoblotting.

SiHa *ADAR1*-KO cells were produced by CRISPR/Cas9 technology. The lentiviral system used was “GSGH11935-Edit-R All-in-one lentiviral sgRNA” (Horizon), where 3 different *ADAR1*-targeting plasmids and 3 lentiviral vectors (GSGH11935-247534211, GSGH11935-247605059, and #GSGH11935-247728949) were pooled together. The plasmids contain both the gene for *Cas9* expression and the gene encoding the RNA guide to target the *Cas9* to the target gene. After purification from bacterial culture, 6 μg plasmid was transfected together with 1.25 μg pVSVG and 3.25 μg psPAX2 plasmids — necessary for lentiviral vector production — into the HEK293T cells using Lipofectamine 2000 Reagent (Invitrogen). After 2 days of incubation at 37°C, the conditioned medium containing the virus was harvested, filtered, and then used to infect SiHa cells by centrifugation. Two infection cycles were applied by using polybrene (MilliporeSigma) at 4 μg/mL for the first and at 8 μg/mL for the second cycle, followed by 2 hours of incubation at 37°C. Infected SiHa cells were then kept in selection with puromycin (1 μg/mL).

### RT-PCR.

RNA was extracted from paraffin-embedded cervical biopsy samples using an RNeasy Plus Mini Kit (QIAGEN, 74134) following deparaffinization with xylene and ethanol. RNA extracted from SiHa and CaSki cells was purified by use of a Total RNA Mini Kit (Geneaid, RB100). After purification, RNAs were treated with DNase I (AMPD1, MilliporeSigma). The following gene-specific Taqman probes were used: *ADAR1* (Hs01017598_g1 and Hs01017601_m1), *IFNbeta1* (Hs01077958_s1), *GAPDH* (Hs02758991_g1) (Thermo Scientific). Additional information is provided in Supplemental Methods.

### Immunoblotting.

Cells were lysed in 50 mM Tris-HCl pH 7.6, 150 mM NaCl, 0.2% Triton X-100, 0.3% NP40, 1 mM EDTA, 50 mM NaF, 1 mM Na_3_VO_4_, 1 mM PMSF, and a cocktail of protease and phosphatase inhibitors (MilliporeSigma). Total protein concentration was determined by Bio-Rad protein assay (BPA). Cell lysates were resolved by SDS-PAGE and blotted to nitrocellulose membranes. Then membranes were blocked with TBS-T (with 0.05% Tween) containing 5% skim milk for 1 hour at room temperature and probed overnight with primary antibodies at 4°C. After washing, secondary anti-rabbit or anti-mouse antibodies (Merck) were incubated for 1 hour at room temperature, and immune-reactive bands were visualized using the ECL chemiluminescence system (Cyanagen) at the iBright FL1500 Imaging System (Thermo Fisher Scientific). The following antibodies were used: anti-ADAR1 (rabbit D7E2M, Cell Signaling Technology); anti–β-actin (AC15, MilliporeSigma); anti–pPKRT446 (rabbit ab32036, Abcam); anti-pPKRT451 (rabbit ab81303, Abcam); anti-PKR antibody (3072, Cell Signaling Technology).

### NK cell assays.

For proliferation, SiHa and CaSki cells were harvested 72 hours after silencing, seeded at a density of 5,000 cells/well in 96-well plates, and stimulated or not with IFN-β (1,000 IU/mL). In some experiments, CM from siADAR1 or siCtr cells was collected at 72–96 hours after transfection and used to stimulate fresh NK cells that had been labeled with the Incucyte Nuclight Reagent (4717, Sartorius) and grown in a 96-well plate (40,000 cells/well) previously coated with 0.01% poly-l-ornithine (MilliporeSigma, P4957). Proliferation was measured by the Incucyte Live-Cell Analysis System, and data were analyzed with Incucyte Zoom software (Sartorius).

NK cell–mediated degranulation was evaluated using the lysosomal marker CD107a (40). Freshly isolated and purified NK cells were cultured for 18 hours in the presence of CM derived from siADAR1 or siCtr cells, then incubated with target cells at an E/T ratio of 1:2. As targets, we used either the same CC-derived cell line from which the CM were collected or K562 cells, the prototypic target for human NK cell–mediated killing. As negative controls, NK cells were incubated with fresh medium (alone or in the presence of target cells). After 4 hours, NK cells were then stained with BD Horizon Fixable Viability Stain 780, anti-CD56/BV421 (562751, BD) and anti-CD107a/APC (560664, BD). In some experiments, NK cells were pretreated with a blocking anti-IFNAR2 (MilliporeSigma, MAB1155) or an isotype control mAb at 37°C for 30 minutes, then incubated with the CM or with IFN-β (100 IU/mL) at 37°C for 18 hours. The mAbs used in the pretreatment were to have a final concentration of 1 mg/mL once NK cells were diluted).

Migration of purified (pNK) or cultured (cNK) NK cells was measured using a Transwell migration chamber (6.5 mm, 5 μm; Costar). As chemoattractant, CM from siADAR1 or siCtr cells was normalized to cell number by dilution with serum-free medium and then added to the lower compartment. After 3–4 hours at 37°C, the migrated cells were counted using a FACSCanto (BD). The percentage of migrated cells was calculated as follows: number of migrated NK cells/number of input NK cells × 100 (41).

### RNA-Seq and bioinformatic analysis.

Bioinformatic analysis on R2, TCGA, CCLE, and DepMap Platforms are described in Supplemental Methods. For RNA-Seq analysis, the library was prepared using 1.5 mcg total RNA, according to the manufacturer’s protocol. Samples of CaSki and SiHa cells were prepared with Kapa RNA HyperPrep with RiboErase (total RNA) and Kapa Globin Depletion Hybridization Oligos (Roche). Samples were sequenced by NovaSeq 6000 Sequencing System, RUN 2 × 101 bp, Illumina Instrument. The average number of reads obtained from each sample was 140 million. Post-sequencing Quality Control was performed by FastQC (https://www.bioinformatics.babraham.ac.uk/projects/fastqc/) v.0.12.1. Adapter sequences were trimmed and low-quality reads removed using cutadapt v3.4. Mapping of RNA and generation of gene counts were done using STAR (42) aligner v.2.7.10b against human reference GRCh38 (build p13) and using feature annotation (v109) downloaded from the Ensembl repository. Differential Expression Analysis of differentially expressed genes (DEGs) was performed by edgeR v.4.0.2; volcano plots and heatmaps were created by ggplot2 v3.4.4 in R (v4.3.3), comparing 2 conditions (siCtr and siADAR). Pathway enrichment and network analyses for DEG lists were performed using clusterProfiler v.4.0 using gene sets from the KEGG pathway database, where small (<10 genes) pathways were removed. In all statistical analysis, an effect was considered statistically significant if the FDR of its corresponding statistical test was ≤5% and considered biologically significant if the logFC was ≥1.

### Organotypic cultures.

3D cultures of ectocervical squamous epithelia equivalents were prepared using a modified version of the protocol previously applied for skin rafts (43). Briefly, collagen rafts were prepared by adding 5 mg/mL rat tail type I collagen (Corning) to DMEM and reconstitution buffer (8:1:1). HFs (1 × 10^6^) were added to 2 mL of the collagen mixture in polycarbonate micron inserts (23 mm, 0.3 μm; Corning) in 6-column deep well plates. The mixture was left to polymerize for 30 minutes at 37°C. After 24 hours, 2 × 10^5^ SiHa or SiHa *ADAR1*-KO cells were seeded on the collagen gel and left to grow for 7 days in CM added in both top and bottom wells. Then organotypic cultures were lifted to the air-liquid interface and cultured for additional 2 weeks in CM. To test the ability of NK cells to infiltrate SiHa layers, 1 × 10^6^ of cultured NK cells were added on top of the organotypic culture in the last 24 hours. Rafts were finally fixed in 10% formalin and embedded in paraffin, and 4 μm slices were stained with H&E or processed for the immunofluorescence procedure. Bright-field images were taken with an Axiocam ICc 5 (Zeiss) connected with an Axioplan 100 microscope (Zeiss). For immunofluorescence, organotypic raft sections were deparaffinized and stained as previously described (44). Primary anti-CD56 mAb (1:50 in PBS; Dako) was incubated for 1 hour at 25°C, followed by goat anti-mouse IgG–Texas red (1:200 in PBS; Jackson Immunoresearch Laboratories) for 30 minutes at 25°C. Nuclei were stained with DAPI (1:1,000 in PBS; MilliporeSigma). For detection and quantification of apoptotic cells, raft sections were processed for TUNEL technology using an In Situ Cell Death Detection Kit (Roche) following the standard protocol. All fluorescence signals were analyzed by scanning cells with an ApoTome System (Zeiss) connected with an Axiovert 200 inverted microscope (Zeiss); image analysis was performed by Axiovision software (Zeiss). Quantitative analysis of TUNEL^+^ nuclei and of CD56^+^ cells was performed by analyzing 10 different microscopy fields randomly taken from 3 independent experiments.

### Statistics.

For IHC staining, quantitative variables are described as mean and range, while qualitative variables are reported as number and percentage. Univariate associations between clinicopathological features and pathological response were evaluated using 1-way ANOVA, χ^2^ test, or Pearson correlation coefficient. Multiple comparisons were performed using univariate ANOVA (2-way ANOVA with Bonferroni’s post hoc test). Some analyses were performed using IBM SPSS Statistics 25. In other analyses, statistical significance between groups was determined by performing 2-tailed Student’s *t* test. Prism 10 (GraphPad) software was used. Graphs show mean values, and error bars represent SD or SEM. *P* values less than 0.05 were considered statistically significant.

### Study approval.

Written informed consent in accordance with the Declaration of Helsinki was obtained from all patients, and approval was obtained from the Ethics Committee of the Sapienza University of Rome (prot. 0372/2023).

### Data availability.

Values for all data points in graphs are reported in the Supporting Data Values file. RNA-Seq data were deposited in the NCBI’s Gene Expression Omnibus database (GEO GSE297095).

## Author contributions

VT and MK conceptualized the study, developed the methodology, performed formal analysis, and wrote the original manuscript draft. SP, MEG, HS, and SR developed the methodology and performed formal analysis. VDM, ML, IP, AP, and FT collected patient samples and clinical information, developed methodology, and performed formal analysis. FB, VM, and DR developed the 3D methodology and performed formal analysis. LLS developed the Incucyte methodology and performed formal analysis. GB, MC, and AZ reviewed and edited the manuscript. CC and AS conceptualized and supervised the study, validated the data, performed formal analysis, acquired funding, administered the project, wrote the original draft, and reviewed and edited the manuscript.

## Supplementary Material

Supplemental data

Unedited blot and gel images

Supporting data values

## Version 1 07/08/2025

Electronic publication
