# Supplementary material for: ADAR1 expression is associated with cervical cancer progression and negatively regulates NK cell activity
Source: JCI Insight. 2025 Jul 8;10(13):e190244. doi: 10.1172/jci.insight.190244 (PMC12288899; doi:10.1172/jci.insight.190244)
Supplement: Supplemental data [file jciinsight-10-190244-s022.pdf]

SUPPLEMENTARY MATERIALS AND METHODS

R2 data analysis

The r2: Genomics Analysis and Visualization Platform (http://r2.amc.nl) was used for analyzing the correlation between *ADAR1* expression (*ADAR*_103) and the overall survival (OS) of patients with CC. The Tumor Cervical Squamous Cell Carcinoma dataset (CESC) (The Cancer Genome Atlas; n=292) was selected for analysis. Expression cutoff: 5759.8945 (min.grp=8). The curves were compared using the log-rank test. *ADAR*1 expression on 10 normal squamous cervical epithelial samples, HSIL, and 21 invasive squamous cell carcinomas, each from different patients, was assayed on single HG_U133A arrays, GEO ID: GSE7803.

TCGA data analysis

Cervical cancer patient data were downloaded from FireBrowse (RNA data:CESC.rnaseqv2__illuminahiseq_rnaseqv2__unc_edu__Level_3__RSEM_genes__data.data.tx, and clinical data: gdac.broadinstitute.org_CESC.Merge_Clinical.Level_1.2016012800.0.0). Genes without a gene name and duplicated names were removed. 306 tumors were present in the data. Log CPM were computed from the data and used for modified Z-score scaling as shown in (1). Normal, Primary Tumor and Metastatic sample types were assigned. The field patient.stage_event.clinical_stage was collapsed into 4 main stages. The *ISG* score is computed as the median expression among the Z-score scaled values for the genes part of the *ISG* list (Table S1).

CCLE analysis

Data for 20 cervical cell lines with positive *ADAR* RNA expression was downloaded from the DepMap Portal (OmicsExpressionProteinCodingGenesTPMLogp1.csv, Model.csv). The RNA data is Z-score scaled as described in (1).

DepMap analysis

The genetic dependency data from the CRISPR screen were extracted from the public data release from the DepMap at the Broad Institute (https://depmap.org/portal/). This database facilitates the construction of a model of cell population dynamics in CRISPR knockout screens, measured using the Chronos score. A Chronos score of <0 indicates a significant role of the gene in the development of the selected cell line. In this study, we downloaded the *ADAR1* CRISPR (DepMap Public 23Q4+Score Chronos) dataset to evaluate the potential role of *ADAR1* in the development of diverse malignant tumors.

Cell lines and primary cultures of human fibroblasts

SiHa (HTB35) cell line was grown in DMEM (Euroclone), whereas CaSki (CRM-CRL-1550) and K562 cells (CCL-243) were cultured in RPMI (EuroClone). All cell lines were from ATCC, and were supplemented with 10% fetal bovine serum (FBS) (Gibco), penicillin, streptomycin, and glutamine (EuroClone). Primary cultures of human fibroblasts (HFs) derived from healthy skin were obtained from patients attending the Dermatology Unit of the Sant'Andrea Hospital of Rome; all patients were adequately and informed and the consent for the biopsy and the subsequent investigation was given and collected in written form, in accordance with guidelines approved by the management of the Sant'Andrea Hospital. HF isolation and culture were performed as previously described (2).

Real-Time (RT) PCR

Reverse transcription was performed on 1 ug of purified RNA using random oligo(dT) and a reaction mix of dNTPs, RNase inhibitor, reverse transcriptase and its buffer (all from Promega). cDNAs were analyzed by RT-PCR, in a reaction mix of 20 µl, containing the Taqman probe, cDNA, and a Master Mix containing dNTPs, buffer solution and Taq polymerase (Applied Biosystem). Gene expression was quantified with the $2^{-\Delta\Delta Ct}$ method. Samples were normalized by comparison with the endogenous constitutively expressed gene *GAPDH*.

References

1. Kung C-P, Cottrell KA, Ryu S, Bramel ER, Kladney RD, Bao EA, et al. Evaluating the therapeutic potential of ADAR1 inhibition for triple-negative breast cancer. Oncogene. 2021;40:189–202.
2. Raffa S, Leone L, Scrofani C, Monini S, Torrisi MR, Barbara M. Cholesteatoma-associated fibroblasts modulate epithelial growth and differentiation through KGF/FGF7 secretion. Histochem Cell Biol. 2012;138:251–69.

SUPPLEMENTARY TABLES

Table S1. List of *ISG*

CCL2
CCL5
CXCL10
CXCL11
CXCL9
IL10
IL12B
IL15
IL15RA
IL18
IFNAR1
IFNGR2
CXCL8
IL23A
IL12A
IL32
IFNB1

Table S2. Patients' characteristics (IHC)

**TOTAL PATIENTS**	***68***
Age (years)	54 (42-66)
LSIL	*17*
HSIL/*in situ* carcinomas (CIS)	*10*
Invasive carcinomas (IC)	*41*
Histotype	
Squamous carcinoma	*34*
Adenocarcinoma	7
Grading	
G1	*3*
G2	16
G3	22

Table S3. Patients' characteristics (fresh biopsies)

**TOTAL PATIENTS**	***82***
Age (years)	51 (21-84)
LSIL	*14*
HSIL/*in situ* carcinomas (CIS)	*10*
Invasive carcinomas (IC)	*18*
Normal mucosa	*40*
Histotype	
Squamous carcinoma	*15*
Adenocarcinoma	3
Grading	
G1	*1*
G2	*9*
G3	*8*
NACT	*8*
Response to NACT	
Progressive disease (PD)	*0*
Stable disease (SD)	*2*
Partial response (PR)	*5*
Complete response (CR)	*1*

Table S4. Reagents used for FACS analysis of fresh biopsies.

REAGENT	SOURCE
Fixable Viability Stain 780, APC-H7	BD Biosciences
Mouse anti-human CD45 antibody, clone HI30, BUV805	BD Biosciences
Mouse anti-human CD3 antibody, clone SK7, BV605	BD Biosciences
Mouse anti-human CD14 antibody, clone M5E2, BV605	BD Biosciences
Mouse anti-human CD19 antibody, clone SJ25C1, BV605	BD Biosciences
Mouse anti-human CD4 antibody, clone RPA-T4, BV605	BD Biosciences
Mouse anti-human CD5 antibody, clone UCHT2, BV605	BD Biosciences
Mouse anti-human CD16 antibody, clone 3G8, BUV737	BD Biosciences
Mouse anti-human CD94 antibody, clone HP-3D9, BV786	BD Biosciences
Mouse anti-human CD56 antibody, clone NCAM16.1, APC R-700	BD Biosciences
Mouse anti-human CD7 antibody, clone M-T701, BUV496	BD Biosciences
Mouse anti-human CD103 antibody, clone Ber-ACT8, BB700/BV480	BD Biosciences
Mouse anti-human CD127 antibody, clone A019D5, APC/Cyanine7	Biolegend
Mouse anti-human Tigit antibody, clone MBSA43, PeFluor610	Invitrogen
Mouse anti-human KIR (CD158a), clone HP-3E4, BV421	BD Biosciences
Mouse anti-human KIR (CD158b), clone CH-L, BV421	BD Biosciences
Mouse anti-human KIR (CD158e1), clone DX9, BV421	BD Biosciences
Mouse anti-human NKp30, clone p30-15, BV650	BD Biosciences
Mouse anti-human Eomes, clone WD1928, PE	Invitrogen
Mouse anti-human T-bet, clone 4B10, PeCy7	Invitrogen
Mouse anti-human Granzyme A, clone CB9, Alexa Fluor 488	Biolegend
Mouse anti-human Granzyme B, clone GB11, Alexa Fluor 647 / BV421	Biolegend
Mouse anti-human Granzyme K, clone G3H69, Alexa Fluor 647 / PerCP-eFluor 710	BD Biosciences
Mouse anti-human RORγT, clone AFKJS-9, Pe-eFluor610	Invitrogen
Mouse anti-human GATA3, clone TWAJ, eFluor660	Invitrogen
Mouse anti-human NKp44, clone P44-8, BUV395	BD Biosciences
Mouse anti-human IL-22, clone 22URTI, PE	Invitrogen
Mouse anti-human IFNγ, clone B27 (RUO), APC	BD Biosciences

SUPPLEMENTARY FIGURES

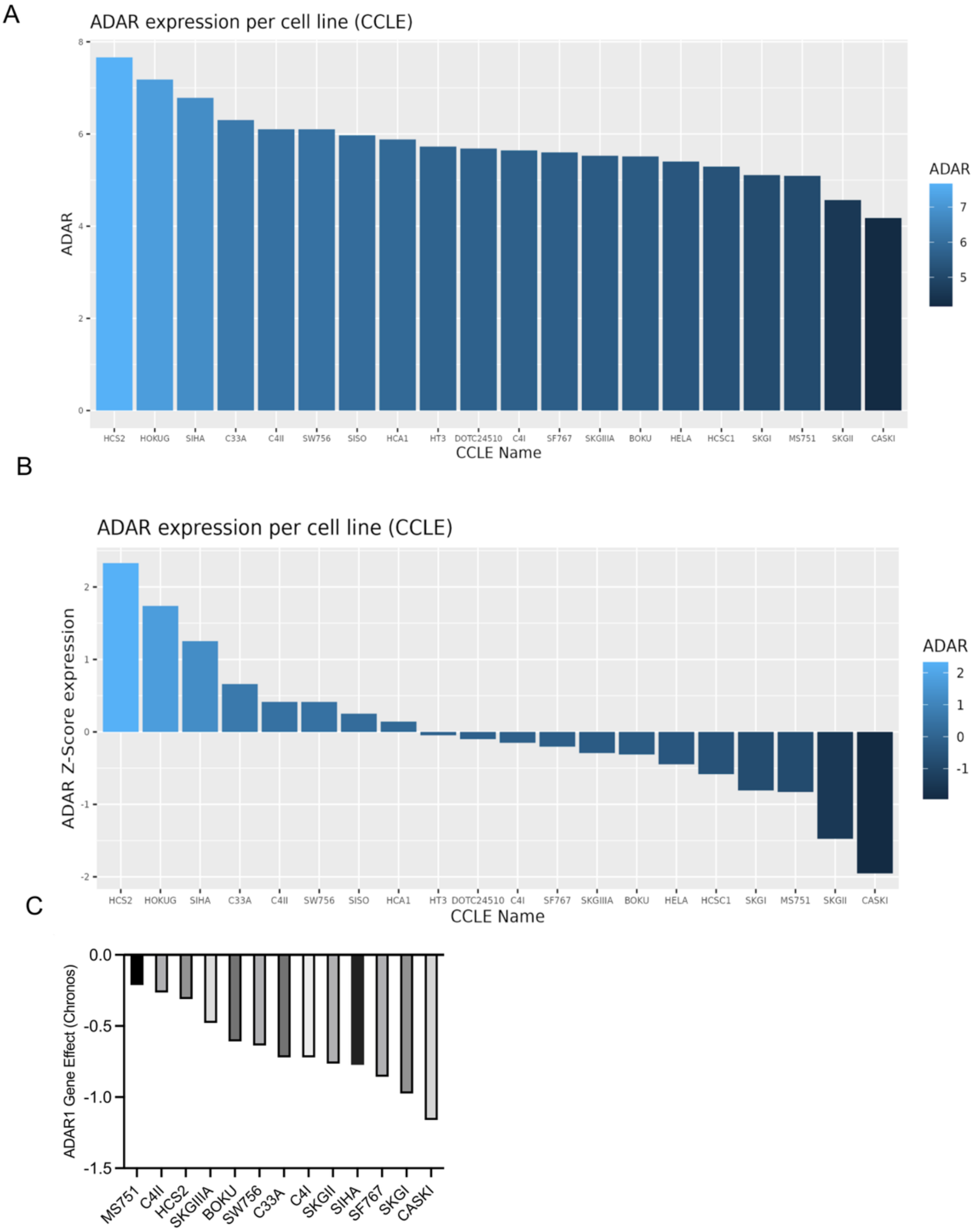

Supplementary Figure 1. *ADAR1* expression in CC-derived cell lines. A, B) Barplots sorted by *ADAR* expression: **A)** raw counts and **B)** Z-score scaled per cell line in the CCLE data. **C)** *ADAR1*-dependency scores in CRISPR knockout screens of different CC-derived cell lines. Lower Chronos scores indicate stronger ADAR1-dependency.

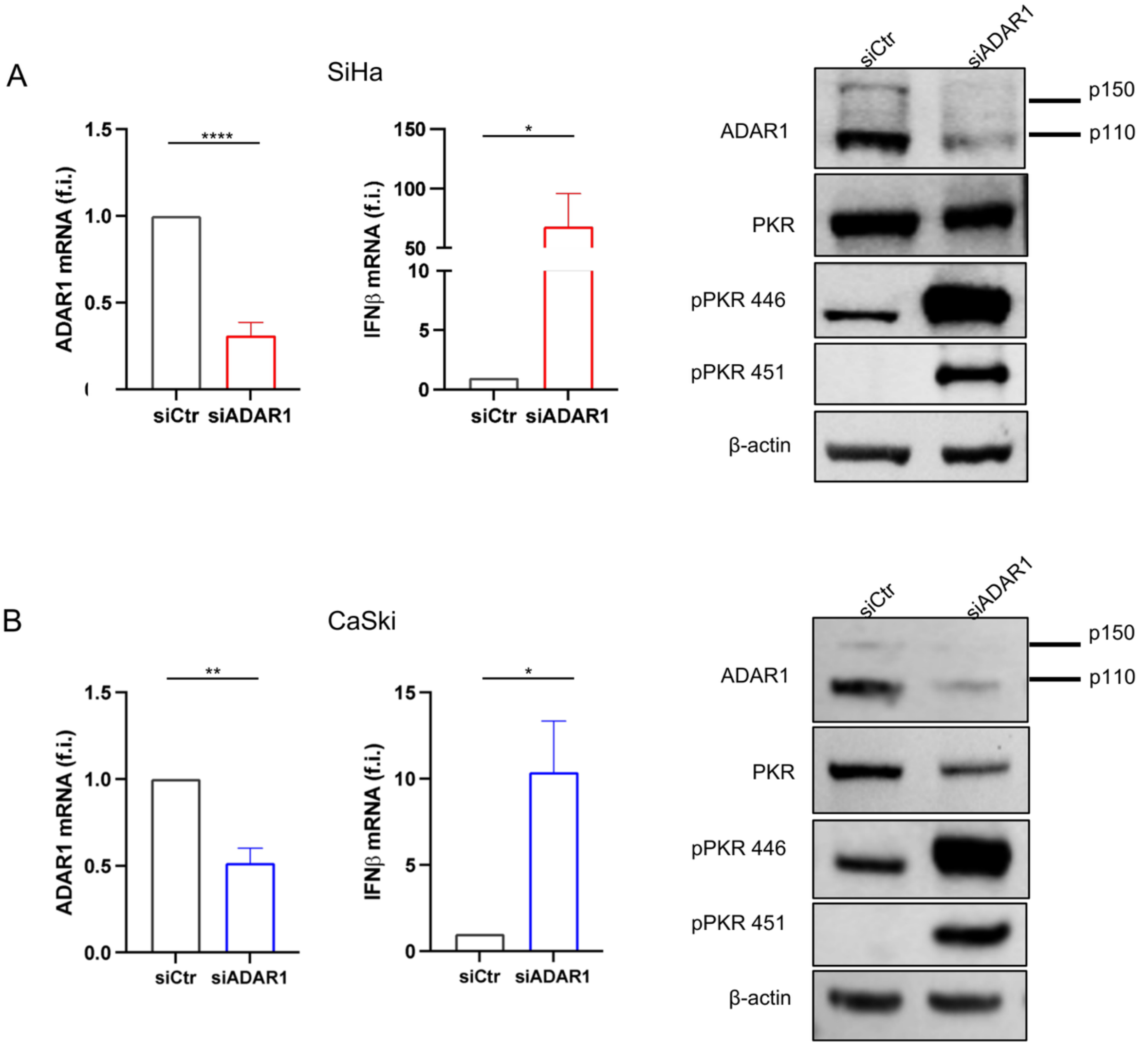

Supplementary Figure 2. ADAR1 silencing promotes *IFN-I* gene expression and activation of the *IFN-I* pathway in SiHa and CaSki CC-derived cell lines. **A)** SiHa or **B)** CaSki cell lines were transfected with an *ADAR1* siRNA (siADAR1) for 72 hrs. RT-PCR and immunoblotting were performed to quantify *ADAR1* mRNA and protein expression, in parallel with *IFNbeta* and phosphorylated PKR expression. *GAPDH* was used as control for RT-PCR. Results derive from 10 (for SiHa) and 5 (for CaSki) independent experiments and from the average of triplicate measurements ± SEM. A representative immunoblot is shown normalized to β-actin. Statistical analysis was performed by paired *t*-test. $*p < 0.05$; $**p < 0.01$; $****p < 0.0001$.

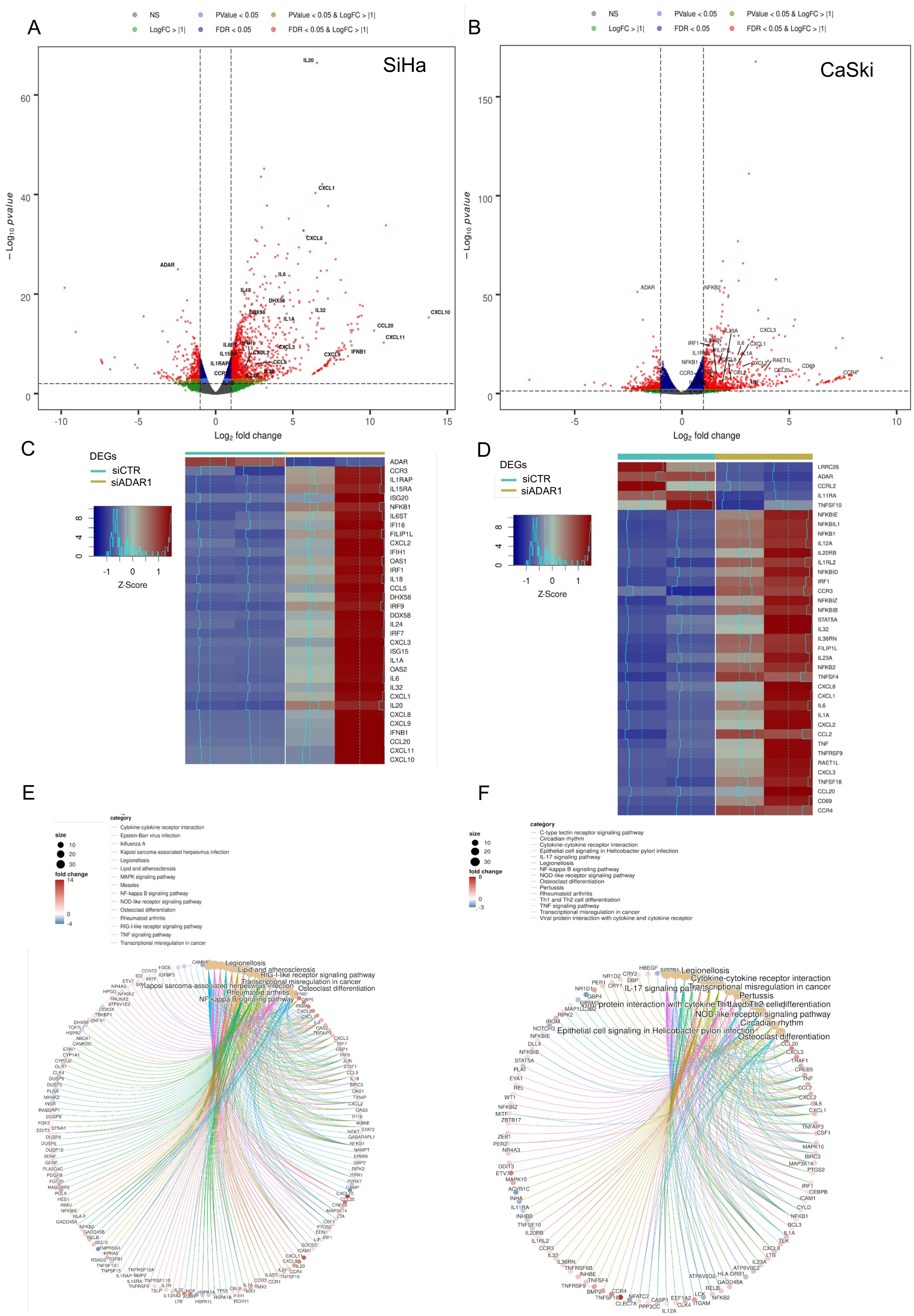

A
NS
LogFC > |1|
PValue < 0.05
FDR < 0.05
PValue < 0.05 & LogFC > |1|
FDR < 0.05 & LogFC > |1|
SiHa
−Log10 pvalue
Log2 fold change
IL20
CXCL1
CXCL8
ADAR
IL6
IL18
DHX58
DDX58
IL32
IL1A
CCL20
CXCL10
CXCL11
IFNB1
CXCL9
CXCL3
CXCL2
CCL5
B
CaSki
ADAR
NFKB2
IL23A
CXCL3
CXCL1
RAET1L
CD69
CCR4
CCL20
TNF
C
DEGs
siCTR
siADAR1
Z-Score
ADAR
CCR3
IL1RAP
IL15RA
ISG20
NFKB1
IL6ST
IFI16
FILIP1L
CXCL2
IFIH1
OAS1
IRF1
IL18
CCL5
DHX58
IRF9
DDX58
IL24
IRF7
CXCL3
ISG15
IL1A
OAS2
IL6
IL32
CXCL1
IL20
CXCL8
CXCL9
IFNB1
CCL20
CXCL11
CXCL10
D
DEGs
siCTR
siADAR1
Z-Score
LRRC26
ADAR
CCRL2
IL11RA
TNFSF10
NFKBIE
NFKBIL1
NFKB1
IL12A
IL20RB
IL1RL2
NFKBID
IRF1
CCR3
NFKBIZ
NFKBIB
STAT5A
IL32
IL36RN
FILIP1L
IL23A
NFKB2
TNFSF4
CXCL8
CXCL1
IL6
IL1A
CXCL2
CCL2
TNF
TNFRSF9
RAET1L
CXCL3
TNFSF18
CCL20
CD69
CCR4
E
category
Cytokine-cytokine receptor interaction
Epstein-Barr virus infection
Influenza A
Kaposi sarcoma-associated herpesvirus infection
Legionellosis
Lipid and atherosclerosis
MAPK signaling pathway
Measles
NF-kappa B signaling pathway
NOD-like receptor signaling pathway
Osteoclast differentiation
Rheumatoid arthritis
RIG-I-like receptor signaling pathway
TNF signaling pathway
Transcriptional misregulation in cancer
size
fold change
F
category
C-type lectin receptor signaling pathway
Circadian rhythm
Cytokine-cytokine receptor interaction
Epithelial cell signaling in Helicobacter pylori infection
IL-17 signaling pathway
Legionellosis
NF-kappa B signaling pathway
NOD-like receptor signaling pathway
Osteoclast differentiation
Pertussis
Rheumatoid arthritis
Th1 and Th2 cell differentiation
TNF signaling pathway
Transcriptional misregulation in cancer
Viral protein interaction with cytokine and cytokine receptor
size
fold change

90

Supplementary Figure 3. ADAR1 silencing in CC-derived cell lines activates the expression of *ISGs* and of proinflammatory cytokines and chemokines and identifies ADAR1-regulated pathways. SiHa (*left panels*) and CaSki (*right panels*) cell lines were transfected with an ADAR1 siRNA for 72 hrs, then RNA was extracted to perform RNA-seq analysis. **A, B)** Volcano-plots. The *x*-axis shows effect size (logarithmized fold-change, FC), and the *y*-axis the negative log10 of *p*-values (-log10(p-value)). The single horizontal dashed line marks the threshold for an unadjusted *p*-value of 0.05. The two vertical dashed lines demarcate the area outside of which there is at least one-fold difference in expression levels between siCtr and siADAR1 cells. The points highlighted in red indicate *ADAR1* and some *ISG*/pro-inflammatory genes. RNA-seq differential gene expression volcano plots display several hundreds of genes up- or down-regulated in both cell lines (see text for details) ($p < 0.05$ and logFC >= |1|). **C, D)** Heatmaps. Selected DEGs of samples were shown as Log counts per million (log cpm). Some up-regulated or down-regulated genes are indicated ($p < 0.05$ and logFC >= |1|). **E, F)** Pathway networks for statistically significant KEGG categories and relationships between genes.

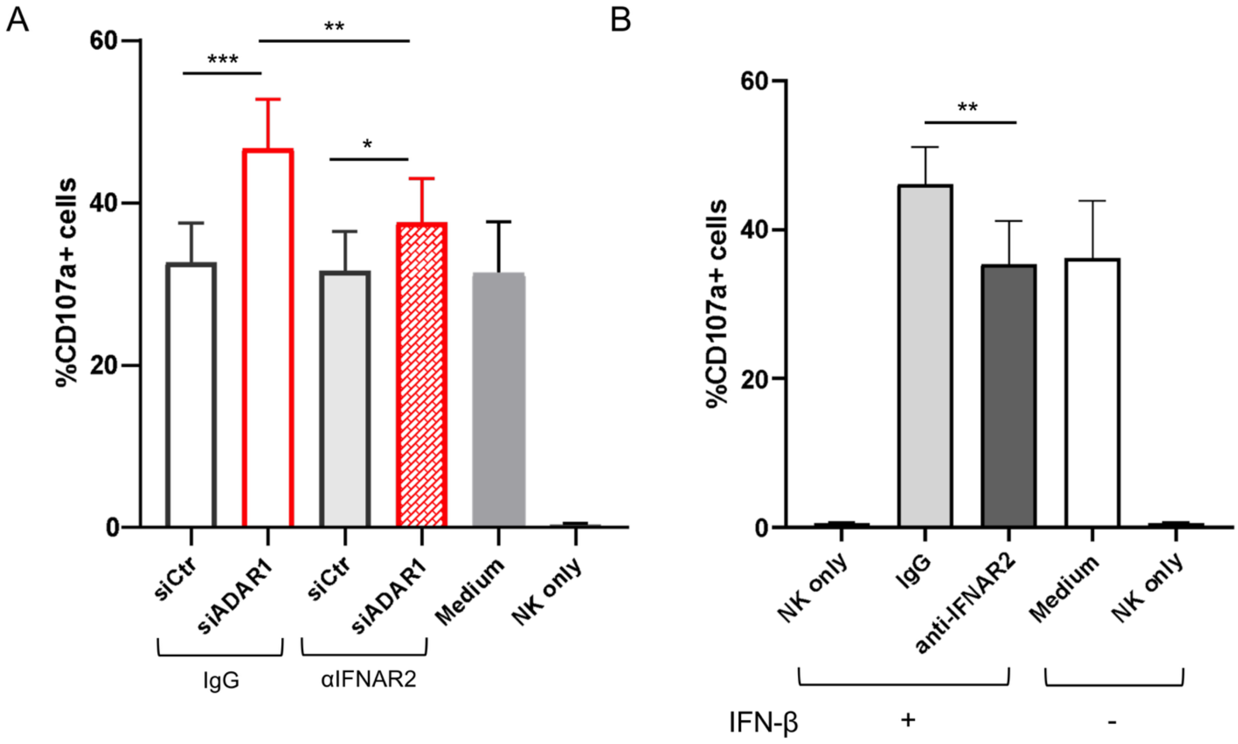

Supplementary Figure 4. Effects of IFN-I on NK cell degranulation upon *ADAR1* silencing. A) Conditioned media from siADAR1 or siCtr SiHa cells was collected at 72 hrs post-transfection and used to stimulate for 18 hrs NK cells, in the presence of a blocking anti-IFNAR2 mAb or of an isotype control IgG. Degranulation against K562 cells used as targets (E:T ratio of 1:2) was evaluated by FACS analysis by CD107a expression on purified NK cells gated as $CD56^{+}$. Pooled data ± SEM are from two independent experiments with NK cells from six different donors. **B)** The same assay was performed on NK cells cultured in the presence of IFNbeta (100 IU/ml, 18 hrs), used as a positive control of the blocking anti-IFNAR2 mAb. Data ± SEM are from NK cells from four different donors. Statistical analysis was performed by paired *t*-test. *$p < 0.05$; **$p < 0.01$; ***$p < 0.001$.

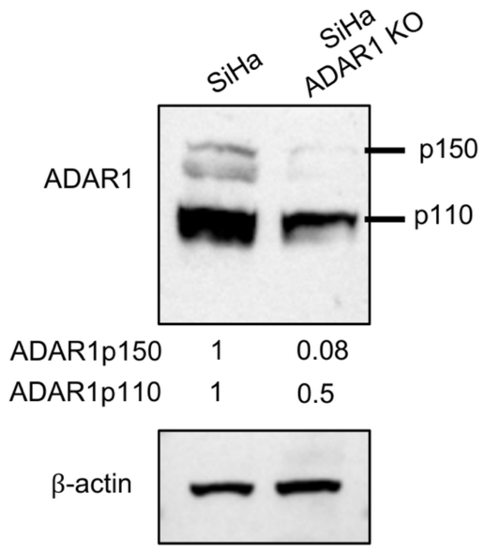

Supplementary Figure 5. Stable ADAR1 silencing in SiHa cell line. CRISPR/Cas9 knock-out for *ADAR1* expression was performed on SiHa cell line (see materials and methods for details). A representative immunoblot to quantify ADAR1 protein expression is shown normalized to β-actin, 14 days after transfection.
