# Supplementary figures and images for: ADAR1 expression is associated with cervical cancer progression and negatively regulates NK cell activity
Source: JCI Insight. 2025 Jul 8;10(13):e190244. doi: 10.1172/jci.insight.190244 (PMC12288899; doi:10.1172/jci.insight.190244)

A

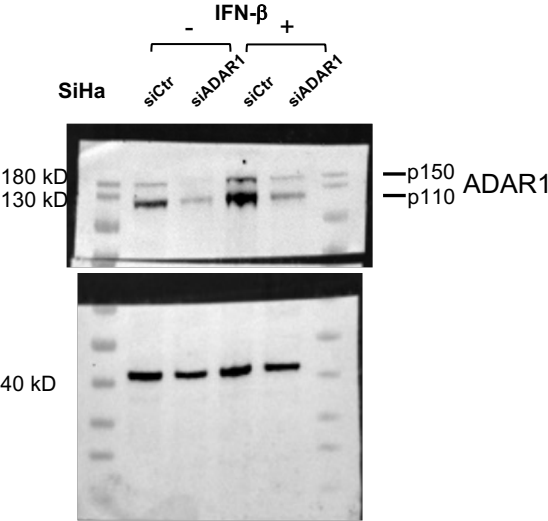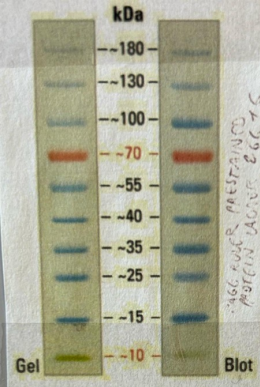

B

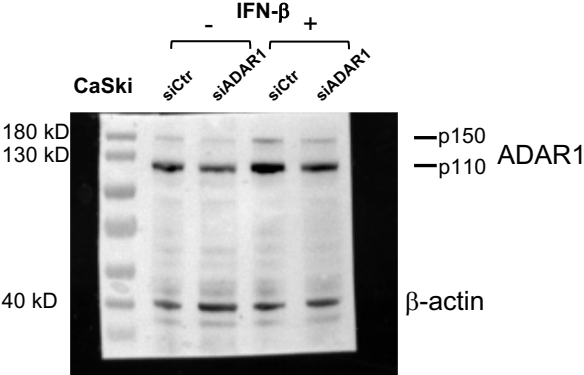

Figure 5

**A**

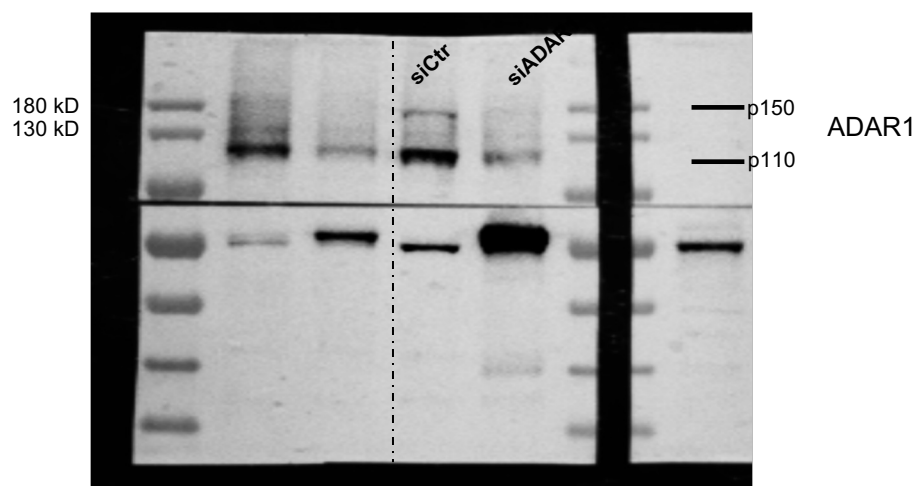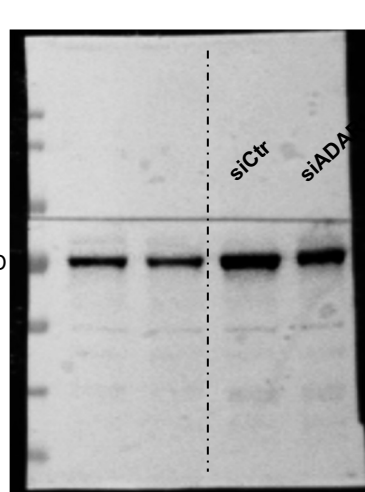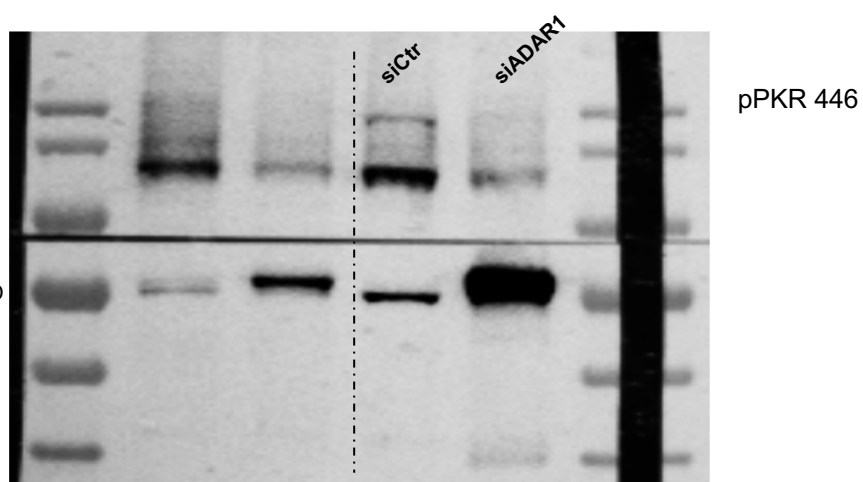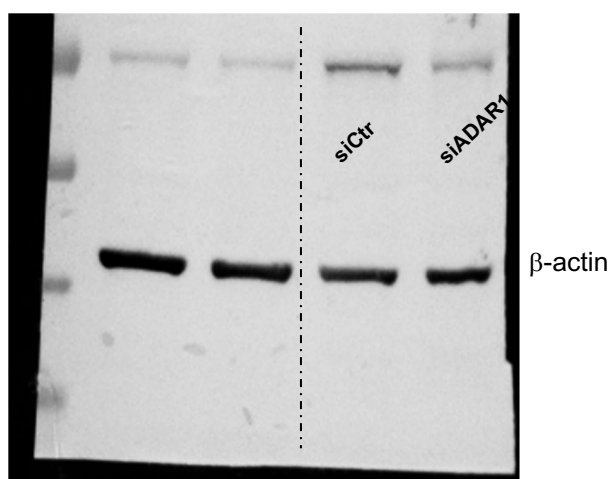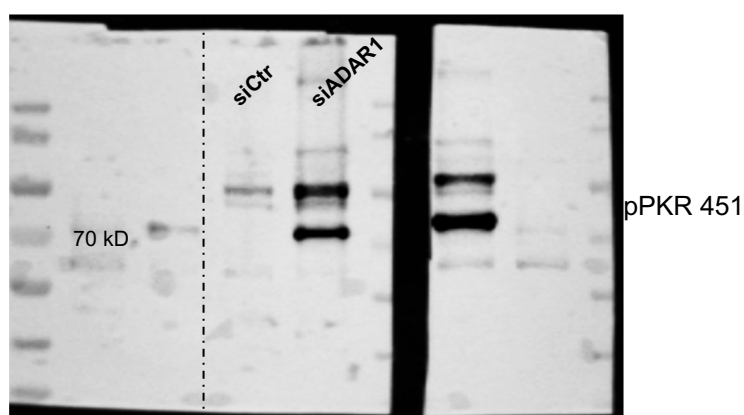

**Figure S2A**

**B**

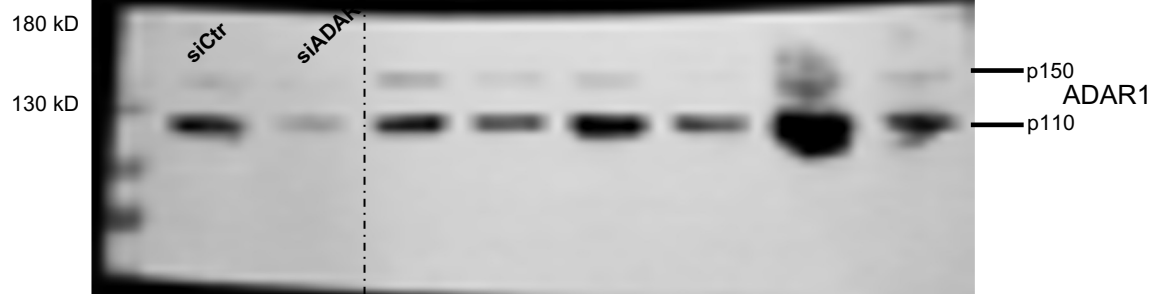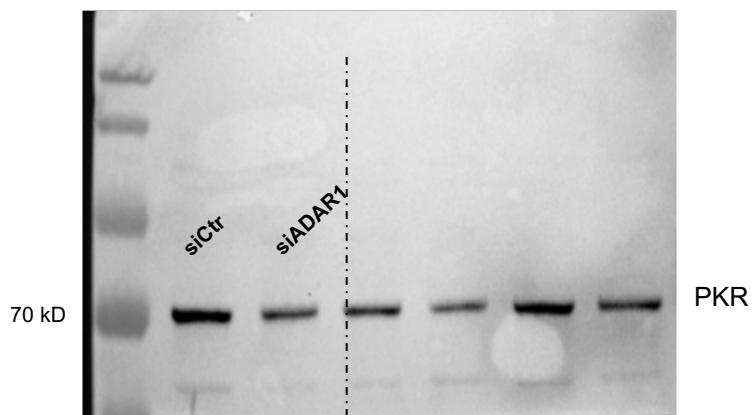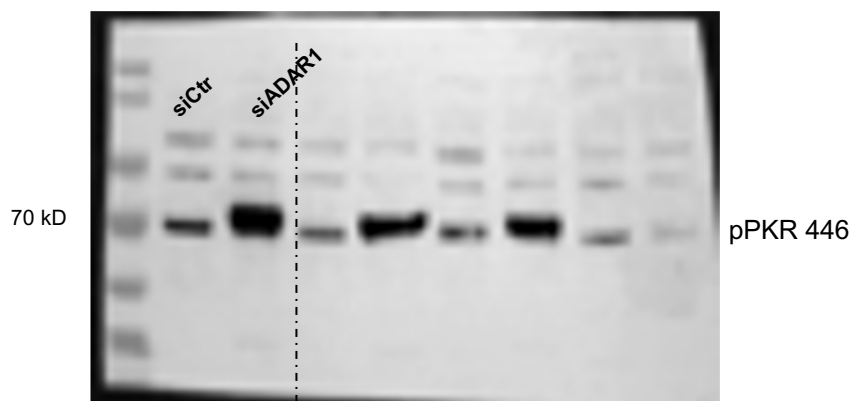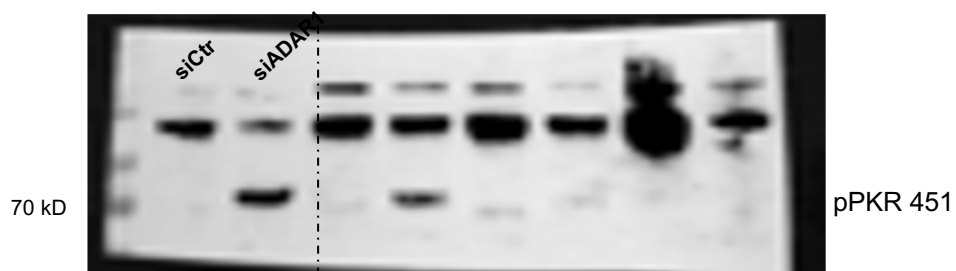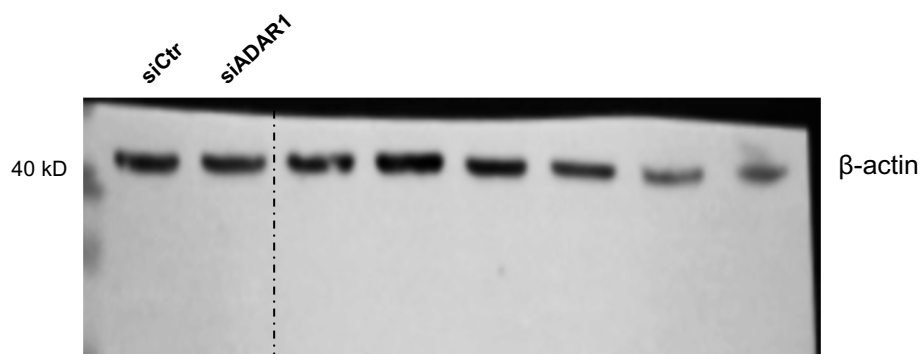

**Figure S2B**

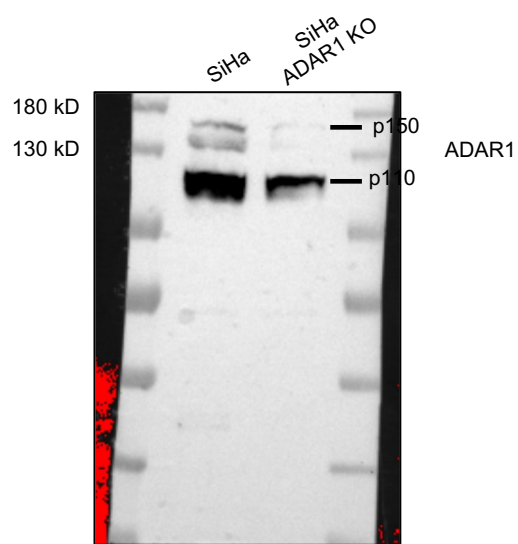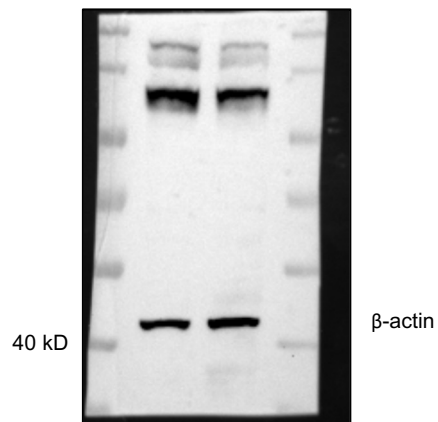

**Figure S5**

Supplement: Unedited blot and gel images [file jciinsight-10-190244-s023.pdf]
